# Supplementary figures and images for: Anemia among pregnant women in Cambodia: A descriptive analysis of temporal and geospatial trends and logistic regression-based examination of factors associated with anemia in pregnant women
Source: PLoS One. 2023 Dec 7;18(12):e0274925. doi: 10.1371/journal.pone.0274925 (PMC10703242; doi:10.1371/journal.pone.0274925)

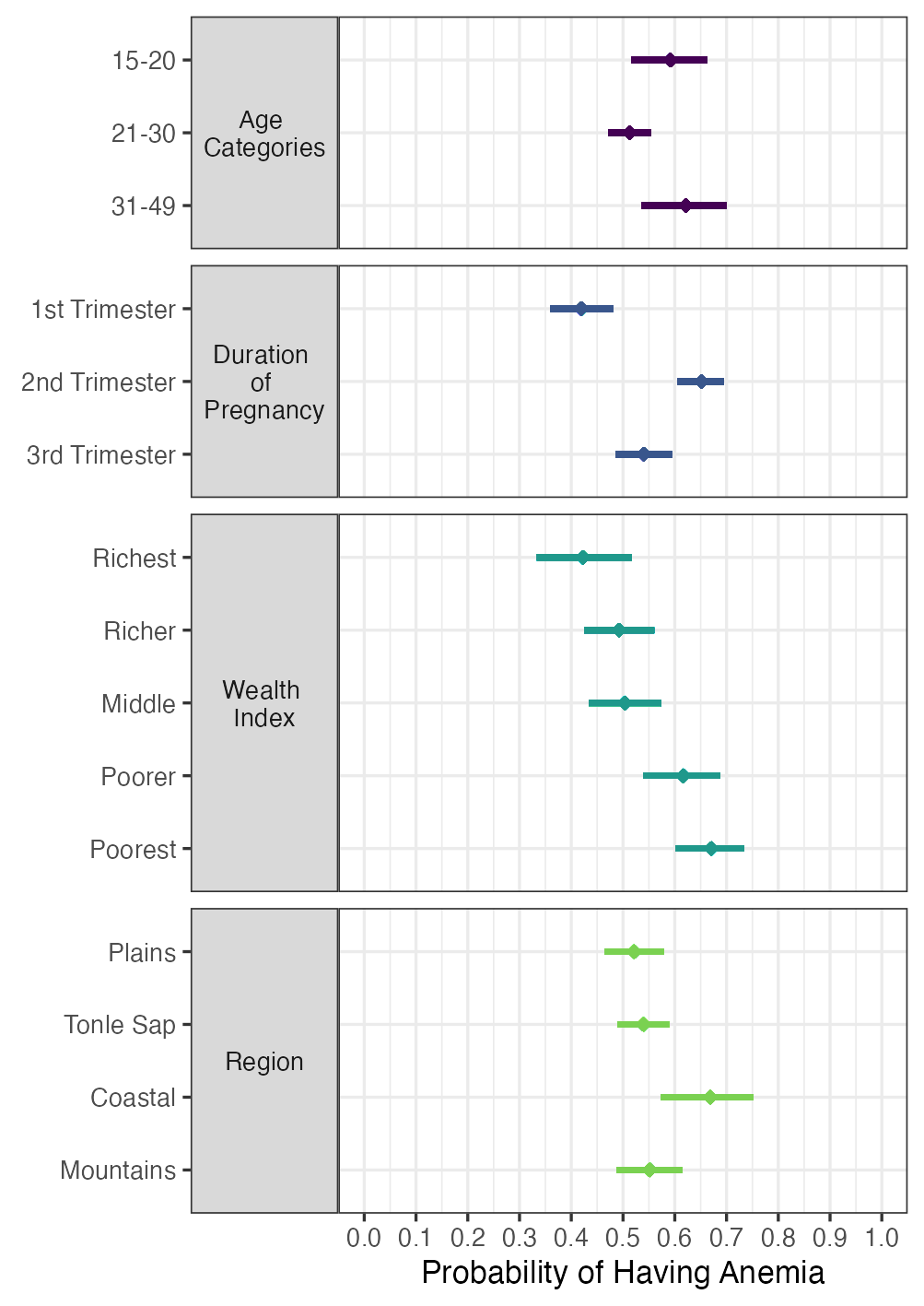

Supplement: S1 Fig — Visualized using “ggplot2” package in R. Probabilities arranged by associated factor; i.e., age, duration of pregnancy, wealth, and region. (TIFF) [file pone.0274925.s001.tiff]
