# Supplementary material for: Anemia among pregnant women in Cambodia: A descriptive analysis of temporal and geospatial trends and logistic regression-based examination of factors associated with anemia in pregnant women
Source: PLoS One. 2023 Dec 7;18(12):e0274925. doi: 10.1371/journal.pone.0274925 (PMC10703242; doi:10.1371/journal.pone.0274925)
Supplement: S1 Table — (DOCX) [file pone.0274925.s002.docx]

**Table S1. Prevalence of Anemia in the 19 domains in CDHS 2005, CDHS 2010 and 2014**

| Province | CDHS 2005 (n=486) | CDHS 2010 (n=467) | CDHS 2014 (n=615) |
| --- | --- | --- | --- |
|  | % of anemia | % of anemia | % of anemia |
| Banteay Meanchey | 62.0 | 37.0 | 30.0 |
| Kampong Cham/Tbong Khmum | 56.0 | 50.0 | 44.0 |
| Kampong Chhnang | 53.0 | 67.0 | 63.0 |
| Kampong Speu | 61.0 | 48.0 | 56.0 |
| Kampong Thom | 62.0 | 57.0 | 62.0 |
| Kandal | 53.0 | 49.0 | 50.0 |
| Kratie | 53.0 | 78.0 | 66.0 |
| Phnom Penh | 34.0 | 31.0 | 46.0 |
| Prey Veng | 50.0 | 60.0 | 69.0 |
| Pursat | 56.0 | 41.0 | 52.0 |
| Siem Reap | 64.0 | 56.0 | 53.0 |
| Svay Rieng | 45.0 | 61.0 | 64.0 |
| Takeo | 64.0 | 40.0 | 26.0 |
| Otdar Meanchey | 78.0 | 30.0 | 59.0 |
| Battambang/Pailin | 55.0 | 58.0 | 50.0 |
| Kampot/Kep | 62.0 | 76.0 | 61.0 |
| Preah Sihanouk/Koh Kong | 76.0 | 48.0 | 65.0 |
| Preah Vihear/Stung Treng | 73.0 | 61.0 | 59.0 |
| Mondul Kiri/Ratanak Kiri | 56.0 | 49.0 | 42.0 |
