# Supplementary material for: Anemia among pregnant women in Cambodia: A descriptive analysis of temporal and geospatial trends and logistic regression-based examination of factors associated with anemia in pregnant women
Source: PLoS One. 2023 Dec 7;18(12):e0274925. doi: 10.1371/journal.pone.0274925 (PMC10703242; doi:10.1371/journal.pone.0274925)

PLOS ONE

Trends and factors associated with anemia among pregnant women aged 15-49 years old in Cambodia: Data analysis of the Cambodia Demographic and Health Surveys

--Manuscript Draft--

**Manuscript Number:**	PONE-D-22-24121
**Article Type:**	Research Article
**Full Title:**	Trends and factors associated with anemia among pregnant women aged 15-49 years old in Cambodia: Data analysis of the Cambodia Demographic and Health Surveys
**Short Title:**	Trends and factors associated with anemia among pregnant women aged 15-49 years old in Cambodia
**Corresponding Author:**	Samnang Um, MPH<br>National Institute of Public Health<br>Phnom Penh, Mountains CAMBODIA
**Keywords:**	Anemia; Cambodia; Pregnant women; Maternal Anemia; CDHS
**Abstract:**	Introduction<br><br>Anemia is a major public health problem affecting roughly 37% (32 million) of pregnant women worldwide. Anemia during pregnancy is a leading cause of low birth weight, preterm birth, and perinatal/neonatal mortality. Women in developing countries are at higher risk of anemia due to micronutrient deficiencies, hemoglobinopathies, infections, or other socio-demographic factors, especially among pregnant women. This study describes the trends and identifies factors associated with anemia among pregnant women aged 15-49 years old in Cambodia.<br><br>Methods<br><br>We analyzed data from the Cambodia Demographic and Health Survey (CDHS) for 2005, 2010, and 2014. Data were pooled across the three survey years for all pregnant women aged 15-49 years. Survey weights were applied to account for the complex survey design of the CDHS. Descriptive statistics were estimated for key sociodemographic characteristics of the study population. We ran bivariate and multivariable logistic regressions to assess factors associated with anemia among pregnant women aged 15-49 years old.<br><br>Results<br><br>Anemia in pregnant women aged 15-49 in Cambodia decreased from 56% in 2005 to 53% in 2014. Compared to pregnant women from the wealthiest households, women from poorest households were more likely to have anemia (AOR = 2.8; 95% CI: 1.76-4.9). Pregnant women from coastal regions were almost twice as likely of having anemia (AOR = 1.9; 95% CI: 1.2-3.0). Pregnant women were more likely anemic if they were in their 2 nd trimester (AOR = 2.6; 95% CI: 1.9-3.6) or 3 rd trimester (AOR = 1.6 95% CI: 1.2-2.3).<br><br>Conclusion<br><br>Anemia remains highly prevalent among pregnant women in Cambodia. Pregnant women from poorer households, those who were further along in the pregnancy, and those living in coastal and sea regions were at greatest risk of anemia. Public health interventions and policies to alleviate anemia should be prioritized and shaped to address these factors.
**Order of Authors:**	Samnang Um, MPH
	Heng Sopheab
	Am Yom
	Jonathan Andrew Muir

Powered by Editorial Manager® and ProduXion Manager® from Aries Systems Corporation

Additional Information:	
**Question**	**Response**
**Financial Disclosure**<br><br>Enter a financial disclosure statement that describes the sources of funding for the work included in this submission. Review the submission guidelines for detailed requirements. View published research articles from *PLOS ONE* for specific examples.<br><br>This statement is required for submission and **will appear in the published article** if the submission is accepted. Please make sure it is accurate.<br><br>**Unfunded studies**<br>Enter: *The author(s) received no specific funding for this work.*<br><br>**Funded studies**<br>Enter a statement with the following details:<br>• Initials of the authors who received each award<br>• Grant numbers awarded to each author<br>• The full name of each funder<br>• URL of each funder website<br>• Did the sponsors or funders play any role in the study design, data collection and analysis, decision to publish, or preparation of the manuscript?<br>• **NO** - Include this sentence at the end of your statement: *The funders had no role in study design, data collection and analysis, decision to publish, or preparation of the manuscript.*<br>• **YES** - Specify the role(s) played.<br><br>* typeset	The author(s) received no specific funding for this work.
**Competing Interests**<br><br>Use the instructions below to enter a competing interest statement for this submission. On behalf of all authors, disclose any competing interests that could be perceived to bias this work—acknowledging all financial support and any other relevant financial or non-financial competing interests.	The authors have declared that no competing interests exist.

This statement is required for submission and **will appear in the published article** if the submission is accepted. Please make sure it is accurate and that any funding sources listed in your Funding Information later in the submission form are also declared in your Financial Disclosure statement.<br><br>View published research articles from *PLOS ONE* for specific examples.<br><br>**NO authors have competing interests**<br><br>Enter: *The authors have declared that no competing interests exist.*<br><br>**Authors with competing interests**<br><br>Enter competing interest details beginning with this statement:<br><br>*I have read the journal's policy and the authors of this manuscript have the following competing interests: [insert competing interests here]*<br><br>* typeset	
**Ethics Statement**<br><br>Enter an ethics statement for this submission. This statement is required if the study involved:<br><br>• Human participants<br>• Human specimens or tissue<br>• Vertebrate animals or cephalopods<br>• Vertebrate embryos or tissues<br>• Field research<br><br>Write "N/A" if the submission does not require an ethics statement.<br><br>General guidance is provided below. Consult the submission guidelines for detailed instructions. **Make sure that all information entered here is included in the**	N/A

Methods section of the manuscript.<br><br>**Format for specific study types**<br><br>**Human Subject Research (involving human participants and/or tissue)**<br>• Give the name of the institutional review board or ethics committee that approved the study<br>• Include the approval number and/or a statement indicating approval of this research<br>• Indicate the form of consent obtained (written/oral) or the reason that consent was not obtained (e.g. the data were analyzed anonymously)<br><br>**Animal Research (involving vertebrate animals, embryos or tissues)**<br>• Provide the name of the Institutional Animal Care and Use Committee (IACUC) or other relevant ethics board that reviewed the study protocol, and indicate whether they approved this research or granted a formal waiver of ethical approval<br>• Include an approval number if one was obtained<br>• If the study involved *non-human primates*, add *additional details* about animal welfare and steps taken to ameliorate suffering<br>• If anesthesia, euthanasia, or any kind of animal sacrifice is part of the study, include briefly which substances and/or methods were applied<br><br>**Field Research**<br><br>Include the following details if this study involves the collection of plant, animal, or other materials from a natural setting:<br>• Field permit number<br>• Name of the institution or relevant body that granted permission	
**Data Availability**<br><br>Authors are required to make all data underlying the findings described fully available, without restriction, and from the time of publication. PLOS allows rare exceptions to address legal and ethical concerns. See the PLOS Data Policy and FAQ for detailed information.	Yes - all data are fully available without restriction

Powered by Editorial Manager® and ProduXion Manager® from Aries Systems Corporation

A Data Availability Statement describing where the data can be found is required at submission. Your answers to this question constitute the Data Availability Statement and **will be published in the article**, if accepted.<br><br>**Important:** Stating 'data available on request from the author' is not sufficient. If your data are only available upon request, select 'No' for the first question and explain your exceptional situation in the text box.<br><br>Do the authors confirm that all data underlying the findings described in their manuscript are fully available without restriction?	
**Describe where the data may be found in full sentences. If you are copying our sample text, replace any instances of XXX with the appropriate details.**<br><br>• If the data are **held or will be held in a public repository**, include URLs, accession numbers or DOIs. If this information will only be available after acceptance, indicate this by ticking the box below. For example: *All XXX files are available from the XXX database (accession number(s) XXX, XXX.*).<br>• If the data are all contained **within the manuscript and/or Supporting Information files**, enter the following: *All relevant data are within the manuscript and its Supporting Information files.*<br>• If neither of these applies but you are able to provide **details of access elsewhere**, with or without limitations, please do so. For example:<br><br>*Data cannot be shared publicly because of [XXX]. Data are available from the XXX Institutional Data Access / Ethics Committee (contact via XXX) for researchers who meet the criteria for access to confidential data.*<br><br>*The data underlying the results*	Our study used 2005, 2010, and 2014 Cambodia Demographic and Health Survey (CDHS) datasets. The DHS data are publicly available from the website: https://dhsprogram.com

presented in the study are available from (include the name of the third party and contact information or URL).<br>• This text is appropriate if the data are owned by a third party and authors do not have permission to share the data.<br><br>* typeset	
Additional data availability information:	

Powered by Editorial Manager® and ProduXion Manager® from Aries Systems Corporation

Manuscript

Click here to access/download;Manuscript;Anemia_Among_Pregnant_Women

Trends and factors associated with anemia among pregnant women aged 15-49 years old in Cambodia: Data analysis of the Cambodia Demographic and Health Surveys

Samnang Um[1*], Heng Sopheab[1], Am Yom[1], Jonathan A. Muir[2]

1 The National Institute of Public Health, Phnom Penh, Cambodia

2 The Global Health Institute, Emory University, Atlanta, Georgia, United States.

*Corresponding author: umsamnang56@gmail.com

Address: Lot#80, 289 Samdach Penn Nouth St. (289), Phnom Penh

ABSTRACT

Introduction

Anemia is a major public health problem affecting roughly 37% (32 million) of pregnant women worldwide. Anemia during pregnancy is a leading cause of low birth weight, preterm birth, and perinatal/neonatal mortality. Women in developing countries are at higher risk of anemia due to micronutrient deficiencies, hemoglobinopathies, infections, or other socio-demographic factors, especially among pregnant women. This study describes the trends and identifies factors associated with anemia among pregnant women aged 15-49 years old in Cambodia.

Methods

We analyzed data from the Cambodia Demographic and Health Survey (CDHS) for 2005, 2010, and 2014. Data were pooled across the three survey years for all pregnant women aged 15-49 years. Survey weights were applied to account for the complex survey design of the CDHS. Descriptive statistics were estimated for key sociodemographic characteristics of the study population. We ran bivariate and multivariable logistic regressions to assess factors associated with anemia among pregnant women aged 15-49 years old.

Results

Anemia in pregnant women aged 15-49 in Cambodia decreased from 56% in 2005 to 53% in 2014. Compared to pregnant women from the wealthiest households, women from poorest households were more likely to have anemia (AOR = 2.8; 95% CI: 1.76-4.9). Pregnant women from coastal regions were almost twice as likely of having anemia (AOR = 1.9; 95% CI: 1.2-3.0). Pregnant women were more likely anemic if they were in their 2nd trimester (AOR = 2.6; 95% CI: 1.9-3.6) or 3rd trimester (AOR = 1.6 95% CI: 1.2-2.3).

Conclusion

Anemia remains highly prevalent among pregnant women in Cambodia. Pregnant women from poorer households, those who were further along in the pregnancy, and those living in coastal and sea regions were at greatest risk of anemia. Public health interventions and policies to alleviate anemia should be prioritized and shaped to address these factors.

Keywords: Anemia, Cambodia, Pregnant Women, Maternal Anemia, CDHS

INTRODUCTION

Anemia during pregnancy occurs in both developed and developing countries and is associated with abnormal outcomes in pregnancy; e.g., greater risk for low birth weight, preterm birth, and perinatal/neonatal mortality [1]. According to the World Health Organization (WHO), anemia is defined as hemoglobin (Hb) levels less than 11.0 g/dl in pregnant women [2]. In 2019, it was estimated that anemia affected approximately 37% (32 million) of pregnant women worldwide , as many as half of all pregnant women in low-income and middle-income countries were diagnosed with anemia [3]. Furthermore, in Southeast Asia countries, 48% of pregnant women were anemia [4]. Data from the Cambodian Demographic Health Survey (CDHS) 2014 suggest that the prevalence of anemia in Cambodia is higher among pregnant women compared with non-pregnant women (i.e., 53.2% compared to 45%) [5].

Anemia in pregnant women has severe consequences on health as well as social and economic development [6]. contributing to low physical activity and increases maternal morbidity and mortality, especially among those with severe anemia [1] and those living in developing countries [7]. The WHO estimates that 20% of maternal deaths are attributable to anemia [2]. In Southeast Asia, half of all maternal deaths were due to anemia in 2016 [8]. In Cambodia, anemia is affected 51.1% of pregnant women aged 15-49 years old in 2019 [9]. Despite significant economic development in Cambodia over the past two decades, the prevalence of anemia in women has not substantially declined.

Pregnant women with anemia are twice as likely to die during or shortly after pregnancy compared to those without anemia [8]. In many countries, anemia varies by socioeconomic factors such as education, household wealth status, occupation, and residence [10]. Pregnant women aged 35 and older are more likely to develop anemia than younger aged pregnant women [11-13]. Risk of anemia

was greater among women living in the lowest wealth quintile, with limited education [14], and rural area [12, 13]. Similarly, repeated childbearing, inadequate water hygiene and sanitation status, and parasitic infection increases the risk of anemia in pregnant women [15]. Factors associated with anemia during pregnancy in developing countries include nutritional deficiencies of iron, vitamin B12, folate, and parasitic diseases (e.g., malaria) [6, 16, 17]. Compared to women who initiated antenatal care (ANC) in the first trimester, the odds of having anemia in pregnancy were significantly higher among pregnant women who initiated ANC in the second trimester (AOR=2.71) and third trimester (AOR=5.01) [18].

The proportion of anemia in Cambodia has slowly decreased in both pregnant and non-pregnant women. To further reduce the prevalence of anemia in Cambodia, improved understanding of its geographical distribution and associated risk factors are required. This enhanced understanding will help identify sub-populations at greater risk for anemia and prioritize geographic areas for targeted interventions. To our knowledge, there are no published peer-reviewed studies that assessed social and demographic factors associated with anemia among pregnant women in Cambodia over time. Hence, we explore temporal and geographic trends of anemia in pregnant women across the 2005, 2010, and 2014 CDHS surveys and identify factors associated with anemia among pregnant women. Understanding these factors may further support policy development and programs with more effective strategies and interventions in Cambodia to reduce the prevalence of anemia among pregnant women and associated health risks such as maternal mortality.

MATERIALS AND METHODS

Data

To analyze trends and factors associated with anemia in pregnant women in Cambodia, we used existing women's data from the 2005, 2010, and 2014 CDHS. The CDHS is a nationally representative population-based household survey that is regularly conducted roughly every 5 years. The survey typically uses two-stage stratified cluster sampling to collect the samples from all provinces that divided into sampling domains. In the first stage, clusters, or enumeration areas (EAs) that represent the entire country are randomly selected from the sampling frame using probability proportional to cluster size (PPS). The second stage then involves the systematic sampling of households listed in each cluster or EA. Interviews were then conducted with women aged 15-49 in selected households. Using the women's survey questionnaire, variables collected by the CDHS include births to women aged 15-49, sociodemographic characteristics, household assets that are used to calculate a household wealth index, health-related indicators and nutritional status, number of ANC visits and other pregnancy/delivery indicators for past births, and involvement in household decision making using questionnaires. It further includes height and weight measurements of women and children, hemoglobin level, malaria test, and vitamin A level [5]. We restricted our sample to pregnant women with recorded hemoglobin level; which resulted in a total sample size of 1,629 pregnant women aged 15-49 (Fig 1).

Fig 1. Selection process and the final sample size from the 2005, 2010, and 2014 CDHS.

Measurements

Outcome variable

Anemia is the outcome variable used in the study. For women in CDHS, hemoglobin was measured by collecting capillary blood from a finger prick with the HemoCue 201+analyzer [19]. The

original variable for anemia level in the CHDS was recorded as a categorical variable with "not anemic", "mild anemia", "moderate anemia", and "severe anemia". Then, the original variable was recoded into the dichotomous variable **Anemia**, with mild, moderate, and severe anemia coded as Anemic = 1 and non-Anemic = 0.

Independent variables

Independent variables included women's and household characteristics. Women's **Age** was categorized into an ordinal level variable with 15-20 years = 1, 21-31 years = 2 (reference category), and 32-49 years = 3. Women's **Marital Status** was coded into a dichotomous variable with Married = 1, Not Married = 0 (includes divorce, single, widowed, and separated). Women's **Education** was coded as an ordinal level variable with Higher = 1 (reference category), Secondary = 2, Primary = 3, No Education = 4. Women's **Occupation** was coded as a categorical variable with Professional = 1 (reference category; included clerical, technical, managerial, professional, sales, and services), Agriculture/Manual = 2 (included skilled and unskilled manual work), Not Working = 3 (1 observation had a missing value). **Wealth Inde**x was coded as an ordinal level variable with richest =1 (reference category), richer = 2, middle = 3, poorer = 4, and poorest = 5 (we opted to use the wealth index that was provided by each respective survey year of the CDHS as opposed to calculating a wealth index across the pooled data. This decision was based on prior research that found wealth status designation was comparable across the CDHS provided indices and an index based on data pooled across years) [20, 21].

Pregnancy Duration (Trimester) was coded as an ordinal level variable with 1st = 1 (*reference category*), 2nd = 2, and 3rd = 3. **BMI** was coded as an ordinal level variable with Overweight or Obese = 1 (reference category), Normal = 2, and Underweight = 3. **Prior Births** was coded as an ordinal level variable with Less than 3 Births = 1 (reference category), 3-5 Births = 2, and 6 or more Births = 3 (3

observations had a missing value). **Tobacco Use** was coded as a dichotomous variable with No Tobacco Use = 0 and Tobacco Use = 1 (possible types of tobacco use included cigarettes, cigars, and chewing tobacco; 2 observations had a missing value).). **Healthcare Barriers** was coded as a dichotomous variable with No Barriers = 0 and 1 or more barriers = 1 (possible barriers reported included distance, money, and waiting time; 1 observation had a missing value).

Residence was a dichotomous variable representing urban vs. rural place of residence with Rural =1, Urban = 0. **Phnom Penh** was a dichotomous variable representing residence in the provincial area surrounding Phnom Penh vs. other regions of residence with Other Regions =1, Phnom Penh = 0. Cambodia's domains/provinces were regrouped for analytic purposes into a categorical variable with 4 geographical **Regions** that were coded as Plains = 1 (reference category), Tonle Sap = 2, Costal/sea = 3, and Mountains = 4. **Health Facility Visit** and **Aborted Pregnancy** were coded as dichotomous variables representing whether the respondent had visited a health facility in the past year or had ever terminated a pregnancy; results are not presented due to lack of statistical significance in bivariate analyses.

Analytic strategy

Data cleaning and analyses were performed using STATA version 16 (Stata Corp 2019, College Station, TX) [22]. Additional visualization was performed using R version 4.2.0 ; the complex survey design of the CHDS was accounted for using the "survey" package [23, 24]. Sample weights and complex survey design were accounted for in both descriptive and logistic regression analyses. To convey temporal and geographic trends in anemia prevalence, weighted estimates of overall and region-specific temporal trends in anemia prevalence were visualized using the ggplot2 package in R [25], a series of

maps illustrating provincial variation in the prevalence of anemia were also prepared using ArcGIS Desktop, Release 10.8.

Bivariate chi-square tests were used to assess significant associations between independent variables of interest including maternal demographic, household characteristics, geographical regions, and health-related) and anemia status. A significant level of any covariates at p-value ≤ 0.15 were included in the multivariate logistic regression analysis [12]. Some factors of interest, such as women's age, residence, geographic regions, survey years, and a total number of children ever born were included based on the literature and prior knowledge regardless of significance levels.

Bivariate logistic regression was used to analyze the magnitude effect of unadjusted associations between anemia and maternal bio-demographic and household characteristics, geographical regions, and health-related factors. Results are reported as Odds Ratios (OR) with 95% confidence intervals (CI). Multivariate logistics regression was then used to assess independent factors associated with anemia after adjusting for other potential confounding factors in the model. Results from the final multivariate model are reported as adjusted odds ratios (AOR) with 95% confidence intervals and corresponding p-values. Results from the final multivariable logistic regression model were considered as statistically significant based on a p-value less than 0.05 and 95% confidence intervals. Multicollinearity were checked for some variables such as education and wealth index, Evaluations of effect medication were not statistically significant. Observations with missing values were omitted from the regression analyses.

This study was approved by the National Ethical Committee for Health Research (Ref: 225 NECHR) Cambodia and the Institutional Review Board (IRB) of ICF in Rockville, Maryland, USA. The CDHS data are publicly accessible and were made available to us upon request to the DHS Program,

ICF. Written consents were obtained from all participants before the interview with participants in the CDHS.

RESULTS

A little over 60% of pregnant women included in the analysis were aged 21-30 years, and almost 98% were married (**Table 1**). Approximately 17% had no schooling, 52% had a primary education, 29% had a secondary education, and only 2.6% had a higher education. Roughly 45% were from poorest or poorer household wealth quintiles. Pregnancy duration was rather evenly distributed with 28% of pregnant women in their 1st trimester, 37% in their 2nd trimester, and 36% in their third trimester. Slightly over 50% of pregnant women had a normal BMI, with another 33% were either overweight or obese. About 80% reported having fewer than 3 prior births. Only about 6% of pregnant women reported using tobacco. A majority (85%) of them resided in rural areas (**Table 1**).

The overall prevalence of anemia among pregnant women decreased slightly from 56% in 2005 to 53% in 2014 (**Fig 2**). This decline varied across the four geographical regions with the highest prevalence of anemia in the coastal region in 2010 (66%). Plain region had the lowest prevalence of anemia across all survey years with prevalence levels in 2005 lower than the prevalence levels of other regions in 2014. Anemia was highest among pregnant women in Preah Vihear and Stung Treng provinces (74.3%), in Kratie province (73%), and in Prey Veng (65.4%) for 2005, 2010 and 2014 respectively (**Fig 3**). Pregnant women living in Phnom Penh had the lowest prevalence of anemia in 2005 and 2010 (i.e., 33.3% in 2005, 30.0% in 2010. In 2014, the lowest prevalence of anemia among pregnant women was observed in Takeo province (23.8%).

Table 1. Factors associated with anemia among study population in bivariate analysis (n=1,567)

Variables		Total		Anemic (n= 846)		Not Anemic (n=721)		p-value
		Frequency	%	Frequency	%	Frequency	%	
**Age (Years)**								
	15-20	305	19.5	182	59.7	123	40.3	
	21-30	951	60.7	474	49.8	477	50.2	**0.002**
	31-49	311	19.8	190	61.1	121	38.9	
**Marital Status**								
	Married	1,533	97.8	824	53.8	709	46.2	0.232
	Not Married	34	2.2	22	64.3	12	35.7	
**Education**								
	Higher	40	2.6	15	36.7	25	63.3	
	Secondary School	449	28.7	221	49.2	228	50.8	**0.025**
	Primary School	820	52.3	461	56.2	359	43.8	
	No Education	259	16.5	150	58.0	109	42.0	
**Occupation**								
	Professional	284	18.1	124	43.7	159	56.3	
	Agricultural/Manual Labor	1,016	64.8	577	56.8	439	43.2	**0.010**
	Not Working	267	17.0	145	54.4	122	45.6	
**Wealth Index**								
	Richest	263	16.8	103	39.0	160	61.0	
	Richer	296	18.9	145	48.9	151	51.1	
	Middle	305	19.5	150	49.0	156	51.0	**< 0.001**
	Poorer	353	22.5	215	60.9	138	39.1	
	Poorest	351	22.4	234	66.9	116	33.1	
**Pregnancy Duration**								
	1st Trimester	432	27.6	182	42.1	250	57.9	
	2nd Trimester	575	36.7	371	64.5	204	35.5	**< 0.001**
	3rd Trimester	561	35.8	294	52.4	267	47.6	
**BMI**								
	Overweight or Obese	516	32.9	254	49.3	262	50.7	
	Normal	888	56.7	518	58.4	370	41.6	**0.006**
	Underweight	159	10.1	72	45.6	86	54.4	
**Prior Births**								
	Less than 3 Births	1,278	81.6	666	52.1	612	47.9	
	3-5 Births	240	15.3	145	60.5	95	40.0	**0.017**
	6 or more Births	49	3.1	35	70.6	14	29.4	
**Tobacco Use**								
	No	1,469	93.7	777	49.7	692	44.2	**0.006**
	Yes	96	6.1	67	3.9	29	2.0	
**Healthcare Barriers**								
	No Barriers	365	23.3	174	47.7	191	52.3	**0.019**
	1 or more Barriers	1,199	76.5	669	55.8	530	44.2	
**Place of Residence**								
	Urban	234	14.9	101	43.2	133	56.8	**0.001**
	Rural	1,333	85.1	745	55.9	588	44.1	
**Phnom Penh**								
	Phnom Penh	389	24.8	634	53.8	545	46.2	0.810
	Other Regions	1,178	75.2	213	54.6	176	45.4	
**Geographic Region**								
	Plains	697	44.5	347	49.7	351	50.3	
	Tonle Sap	525	33.5	290	55.4	234	44.6	**0.019**
	Coastal	99	6.3	64	64.8	35	35.2	
	Mountains	246	15.7	144	58.7	102	41.3	
**Survey Year**								
	2005	486	31.0	272	56.0	214	44.0	
	2010	467	29.8	247	52.9	220	47.1	0.696
	2014	615	39.2	327	53.2	288	46.8	

Plains: Phnom Penh, Kampong Cham/Tbong Khmum, Kandal, Prey Veng, Svay Rieng, and Takeo; **Tonle Sap:** Banteay Meanchey, Kampong Chhnang, Kampong Thom, Pursat, Siem Reap, Battambang, Pailin, and Otdar Meanchey; **Coastal/sea:** Kampot, Kep, Preah Sihanouk, and Koh Kong; **Mountains:** Kampong Speu, Kratie, Preah Vihear, Stung Treng, Mondul Kiri, and Ratanak Kiri. Variables with missing values for fewer than 3 observations include: Occupation, BMI, Tobacco Use, and Healthcare Barriers.

Fig 2. Overall and Regional Trends of Anemia among Pregnant Women by Survey Year

Fig 3. Geographical distribution of anemia among pregnant women aged 15-49 years old.

Regarding socio-demographic factors associated with anemia in pregnancy in logistic regression analysis, pregnant women were more likely to have anemia if they were aged 15-20 (OR = 1.5; 95% CI: 1.1-2.0) or aged 31-49 (OR = 1.6; 95% CI: 1.1-2.2) compared to pregnant women aged 21-30 (**Figure 4**). Pregnant women with no formal education were more likely to have anemia compared to women with a higher education (2.4; 95% CI: 1.1-5.1) as were women with only a primary level education (OR = 2.2; 95% CI: 1.1-4.5). Women working in agricultural or manual labor fields were more likely to have anemia compared to women working in professional fields (OR = 1.7; 95% CI: 1.2-2.3). Juxtapose pregnant women from the richest wealth quintile, pregnant women with lower wealth were more likely to have anemia; e.g., women from the poorest quintile were over 3 times as likely to have anemia (OR = 3.2; 95% CI: 2.1-4.7) and women from the poorer quintile were over 2 times as likely to have anemia (OR = 2.4; 95% CI: 1.6-3.6).

Health indicators associated with anemia in bivariate analysis included pregnancy duration, BMI, number of prior births, tobacco use, and barriers to healthcare. Pregnant women were more likely to have anemia if they were in their 2nd trimester (OR = 2.5; 95% CI: 1.8-3.4) or their 3rd trimester (OR = 1.5; 95% CI: 1.1-2.1) compared to pregnant women in their 1st trimester. A normal BMI was also associated with anemia during pregnancy compared to a BMI indicating that a pregnant woman was overweight or obese (OR = 1.5; 95% CI: 1.1-1.9). Compared to having had fewer than 3 births, pregnant women with 6 or more prior births were more likely to have anemia (2.2; 95% CI: 1.2-4.2). Pregnant

women using 1 type of tobacco were more likely to have anemia compared to pregnant women not using tobacco (1.9; 95% CI: 1.1-3.2); using 2 or more types of tobacco was likewise positively associated with having anemia (17.5; 95% CI: 3.7-83.3). Finally, reporting 1 or more barriers to accessing healthcare was associated with anemia during pregnancy (1.4; 95% CI: 1.1-1.8).

Fig 4. Factors associated with anemia among pregnant women aged 15-49 years old in bivariate logistic regression analysis (n=1,567).

Geographic regions of residence were likewise associated with a woman having anemia during pregnancy. Pregnant women from rural areas were more likely to have anemia compared to pregnant women from urban areas (1.7; 95% CI: 1.3-2.2). Likewise, pregnant women living outside of the area surrounding Phnom Penh were more likely to have anemia compared to pregnant women from the Phnom Penh area (1.6; 95% CI: 1.1-2.3). Coastal regions were positively associated with anemia compared to plains regions (1.9; 95% CI: 1.2-2.9) as were mountain regions (1.43; 95% CI: 1.04-1.98).

In the final multivariate logistic model (**Figure 5**), factors independently associated with anemia included age, wealth index, pregnancy duration, BMI, and geographical regions. Pregnant women were more likely to have anemia if they were aged 31-49 (AOR = 1.6; 95% CI: 1.0-2.3) compared to pregnant women aged 21-30. Compared to pregnant women from the richest wealth quintile, pregnant women from the poorest quintile were almost 3 times as likely to have anemia (AOR = 2.8; 95% CI: 1.76-4.9) as were women from the poorer quintile (AOR = 2.2; 95% CI: 1.3-3.9). Pregnant women were more likely anemic if they were in their 2nd trimester (AOR = 2.6; 95% CI: 1.9-3.6) or 3rd trimester (AOR = 1.6 95% CI: 1.2-2.3) compared to their 1st trimester. Pregnant women with a normal BMI were more likely of developing anemia (AOR = 1.4; 95% CI: 1.0-1.8) compared to pregnant women who were

overweight or obese. Pregnant women living in coastal regions had higher odds having anemia (AOR = 1.9; 95% CI: 1.2-3.0) compared to those living in plain region. Statistically significant associations were further visualized as predicted probabilities (see Figures 6-9 in the supplement).

Fig 5. Factors independently associated with anemia among pregnant women aged 15-49 years old in multivariate logistic regression analysis (n=1,567).

DISCUSSION

Prevalence of anemia among pregnant women in Cambodia decreased slightly from 56% in 2005 to 53% in 2014. This prevalence level was relatively higher than many Southeast Asian countries during the same time period [26, 27]; e.g., in Thailand the prevalence of anemia among pregnant women ranged from 31% in 2005 to 31.9% in 2016 and prevalence of anemia among pregnant women in Vietnam ranged from 33.8% in 2005 to 28.2% in 2015. The elevated prevalence of anemia among pregnant women in Cambodia may be explained by dietary habits, deficiencies in micronutrients such as iron or vitamin A, hemoglobinopathy, malaria infections, and helminths [16]. Interventions to help mitigate anemia in Cambodia include the promotion of a diversified diet; recommendations that all pregnant women attend at least 4 ANC visit, receive a standard dose of 30–60 mg iron and 400 μg folic acid beginning as soon as possible during gestation, and iron-containing supplements no later than the first trimester of pregnancy; prevention and treatment of malaria; use of insecticide-treated bed nets; helminth prevention and control; delayed cord clamping; and increased birth spacing [28]. However, some have noted that the factors addressed by these interventions cannot fully account for the elevated prevalence of anemia [29], hence the interest in better understanding associations between anemia and

demographic, economic, and social factors as well as health-seeking behaviors across different parts of the country [30].

Our results suggest independent associations between anemia in pregnant women and age, wealth, pregnancy duration, BMI, and region of residence. Older aged pregnant women were more likely to have anemia compared to young pregnant women, which is consistent with previous studies [11-13] and may reflect biological and physical disadvantages with increased maternal age; e.g., developing gestational diabetes, high blood pressure during pregnancy, a mother having multiple pregnancies, and enduring the collective impact of exhausting labor-related complications [31-33]. Consistent with studies from Rwanda [34], Ethiopia [35], and Uganda [36], pregnant women were also more likely to have anemia if they were from the poorest wealth quintiles with the patterns of association between anemia and wealth exhibiting a dose response to increased poverty. Lower socioeconomic status is often associated with negative consequences regarding consumption of a healthy diet, greater chance of infection diseases, and less access to healthcare services [38]. In contrast, individuals from higher socioeconomic groups have a comparative advantage that enables them to purchase sufficient food with greater variety and quality, thus protecting against anemia [39]. In addition, pregnant women living in the coastal regions of residence were more likely have anemia compared to women living in plains regions. These differences in the prevalence of anemia might be due to variations in socioeconomic status, attention given to focused antenatal care and supplementation of iron sulfate throughout the pregnancy, dietary patterns, sample size, and geographical and lifestyle variations. Finally, pregnant women with a normal BMI were more likely of have anemia compared to pregnant women who were overweight or obese, which is surprising given

prior studies suggesting that higher BMI is negatively associated with iron status among pregnant women [40]. This association should be evaluated further in the future.

Hard to see a trend when 3 points are used !!.

This study was conducted using pooled Demographic and Health surveys for 2005, 2010, and 2014, which enabled us to evaluate temporal and geographical trends in anemia among pregnant women using nationally representative data and generalize our findings to pregnant women throughout Cambodia. However, using cross-sectional data limits our ability to assess causal relationship among the associated factors that we identified. Using secondary data that was gathered to collect health and demographic data across a broad variety of variables related to child and maternal health enabled us to evaluate some of the factors likely associated with anemia among pregnant women, but other factors such as diet, nutritional supplements (Vitamin B12, folate); malaria infection, non-communicable diseases such as hypertension and diabetes, and awareness about anemia were excluded from the analysis due to data limitations. Finally, the most recent round of CDHS data was gathered in 2014, which may no longer reflect present prevalence of anemia among pregnant women in Cambodia. Future research should further evaluate these relationships and the general prevalence of anemia among pregnant Cambodian women once additional data becomes available (data gathering for the next round of Cambodia DHS data is underway, but delayed due to COVID-19).

Our findings suggest that the high prevalence of anemia among pregnant women in Cambodia declined only slightly over the 10-year period between 2005 and 2014. This is concerning given that anemia during pregnancy is a public health problem that should be actively addressed to mitigate the proximal and distal health risks associated with the condition. We identified age, socioeconomic status, higher BMI, and regions of residence as potential risk factors associated with anemia during pregnancy. Public health practitioners and policy makers should consider demographic groups represented by

these characteristics for better targeting interventions to address anemia among pregnant women in Cambodia.

ACKNOWLEDGMENTS

The authors would like to thank the National Institute of Public Health for allowing us to take part in Sabbatical for Writing Program 2022. We also sincerely thank the DHS-ICF, who approved the data used for this paper.

AUTHORS' CONTRIBUTIONS

Samnang Um contributed to the conceptualization, methodology, data analysis, writing the original draft, review, and editing; **Heng Sopheab** contributed to writing, review, and editing the manuscript; **Am Yom** contributed to writing, review, and editing the manuscript; and **Jonathan A. Muir** contributed to methodology, data analysis, writing, review, and editing the manuscript. All authors read and approved the final manuscript.

DATA AVAILABILITY

Our study used 2005, 2010, and 2014 Cambodia Demographic and Health Survey (CDHS) datasets. The DHS data are publicly available from the website: https://dhsprogram.com

FUNDING

The authors had no funding received for this study

COMPETING INTERESTS

The authors have declared that no competing interests exist

ABBREVIATIONS

ANC, Antenatal Care; AOR, Adjusted Odds Ratio; CDHS, Cambodian Demographic Health Survey; EA, Enumeration areas; Hb, Hemoglobin; WHO, World Health Organization

REFERENCES

1. Rahman MM, Abe SK, Rahman MS, Kanda M, Narita S, Bilano V, et al. Maternal anemia and risk of adverse birth and health outcomes in low-and middle-income countries: systematic review and meta-analysis, 2. The American journal of clinical nutrition. 2016;103(2):495-504.
2. WHO. The global prevalence of anaemia in 2011. Geneva: World Health Organization. 2015.
3. World Health Organization, Anaemia in women and children. Available at: https://www.who.int/data/gho/data/themes/topics/anaemia_in_women_and_children. Accessed on April 2, 2022.
4. WHO U. Global Nutrition Targets 2025: Breastfeeding policy brief (WHO/NMH/NHD14. 7). Geneva: World Health Organization. 2014.
5. NIS. National Institute of Statistics. Directorate General for HC. International ICF . Cambodia Demographic and Health Survey 2014. Phnom Penh, Cambodia: National Institute of Statistics/Cambodia, Directorate General for Health/Cambodia, and ICF International; 2015. 2014.
6. McLean E, Cogswell M, Egli I, Wojdyla D, De Benoist B. Worldwide prevalence of anaemia, WHO vitamin and mineral nutrition information system, 1993–2005. Public health nutrition. 2009;12(4):444-54.
7. Elzahrani S. Prevalence of iron deficiency anemia among pregnant women attending antenatal clinics at Al-Hada Hospital. Canadian Journal on Medicine. 2012;3(1):10-4.
8. Daru J, Zamora J, Fernández-Félix BM, Vogel J, Oladapo OT, Morisaki N, et al. Risk of maternal mortality in women with severe anaemia during pregnancy and post partum: a multilevel analysis. The Lancet Global Health. 2018;6(5):e548-e54.
9. Bank W. Cambodia KH: Prevalence of Anemia among Pregnant Women: %; [https://www.ceicdata.com/en/cambodia/health-statistics/kh-prevalence-of-anemia-among-pregnant-women-]. 2019.
10. Balarajan Y, Ramakrishnan U, Özaltin E, Shankar AH, Subramanian S. Anaemia in low-income and middle-income countries. The lancet. 2011;378(9809):2123-35.
11. Woldegebriel AG, Gebregziabiher Gebrehiwot G, Aregay Desta A, Fenta Ajemu K, Berhe AA, Woldearegay TW, et al. Determinants of anemia in pregnancy: findings from the Ethiopian health and demographic survey. Anemia. 2020;2020.
12. Lin L, Wei Y, Zhu W, Wang C, Su R, Feng H, et al. Prevalence, risk factors and associated adverse pregnancy outcomes of anaemia in Chinese pregnant women: a multicentre retrospective study. BMC pregnancy and childbirth. 2018;18(1):1-8.
13. Wu Y, Ye H, Liu J, Ma Q, Yuan Y, Pang Q, et al. Prevalence of anemia and sociodemographic characteristics among pregnant and non-pregnant women in southwest China: a longitudinal observational study. BMC Pregnancy and ChildBirth. 2020;20(1):1-10.
14. Sunuwar DR, Singh DR, Chaudhary NK, Pradhan PMS, Rai P, Tiwari K. Prevalence and factors associated with anemia among women of reproductive age in seven South and Southeast Asian countries: Evidence from nationally representative surveys. PloS one. 2020;15(8):e0236449.
15. Harding KL, Aguayo VM, Namirembe G, Webb P. Determinants of anemia among women and children in Nepal and Pakistan: An analysis of recent national survey data. Maternal & child nutrition. 2018;14:e12478.
16. Charles CV, Summerlee AJ, Dewey CE. Anemia in Cambodia: Prevalence, etiology and research needs. Asia Pacific journal of clinical nutrition. 2012;21(2):171-81.
17. Charles CV, Dewey CE, Daniell WE, Summerlee AJ. Iron-deficiency anaemia in rural Cambodia: community trial of a novel iron supplementation technique. European journal of public health. 2011;21(1):43-8.
18. Tibambuya BA, Ganle JK, Ibrahim M. Anaemia at antenatal care initiation and associated factors among pregnant women in West Gonja District, Ghana: a cross-sectional study. Pan Afr Med J. 2019;33:325. Epub 2019/11/07. doi: 10.11604/pamj.2019.33.325.17924. PubMed PMID: 31692871; PubMed Central PMCID: PMCPmc6815505.
19. Croft T, Marshall A, Allen C. Guide to DHS Statistics. Rockville, Maryland, USA: ICF; 2018. 2018.

20. Muir JA. Societal Shocks as Social Determinants of Health: The Ohio State University; 2021.
21. Muir JA. Another mHealth? Examining Motorcycles as a Distance Demolishing Determinant of Health Care Access in South and Southeast Asia. Journal of Transport & Health. 2018;11:153-66.
22. Cunningham SA, Muir JA. Data Cleaning. In: Nichols AN, Edlund J, editors. The Cambridge Handbook of Research Methods and Statistics for the Social and Behavioral Sciences: Volume 1: Building a Program of Research. 1: Cambridge University Press; 2023.
23. R Core Team. R: A Language and Environment for Statistical Computing. Vienna, Austria: R Foundation for Statistical Computing; 2022.
24. Lumley T. Package 'survey'. Available at the following link: https://cran r-project org. 2020.
25. Wickham H. ggplot2: elegant graphics for data analysis: springer; 2016.
26. Our World in Data. Prevalence of anemia in pregnant women, 2000 to 2019 Available at: https://ourworldindata.org/grapher/prevalence-of-anemia-in-pregnant-women?tab=chart. Accessed on March 26, 2022.
27. World Bank. Prevalence of anemia among pregnant women (% of women ages 15–49). Available at: https://data.worldbank.org/indicator/SH.PRG.ANEM. Accessed on March 26, 2022.
28. Barrett B. Nutrition Profile of Cambodia: A Review of Current Conditions and Strategies to Prevent Macronutrient and Micronutrient Deficiencies. 2020.
29. Wieringa FT, Dahl M, Chamnan C, Poirot E, Kuong K, Sophonneary P, et al. The High Prevalence of Anemia in Cambodian Children and Women Cannot Be Satisfactorily Explained by Nutritional Deficiencies or Hemoglobin Disorders. Nutrients. 2016;8(6):348. PubMed PMID: doi:10.3390/nu8060348.
30. Charles CV, Dewey CE, Hall A, Hak C, Channary S, Summerlee AJ. Anemia in Cambodia: A cross-sectional study of anemia, socioeconomic status and other associated risk factors in rural women. Asia Pacific journal of clinical nutrition. 2015;24(2):253-9.
31. Press MC. Pregnancy after 35: Healthy moms, healthy babies. Available at: https://www.mayoclinic.org/healthy-lifestyle/getting-pregnant/in-depth/pregnancy/art-20045756. Accessed on March 31, 2022. 2022.
32. Londero AP, Rossetti E, Pittini C, Cagnacci A, Driul L. Maternal age and the risk of adverse pregnancy outcomes: a retrospective cohort study. BMC pregnancy and childbirth. 2019;19(1):1-10.
33. Cavazos-Rehg PA, Krauss MJ, Spitznagel EL, Bommarito K, Madden T, Olsen MA, et al. Maternal age and risk of labor and delivery complications. Maternal and child health journal. 2015;19(6):1202-11.
34. Habyarimana F, Zewotir T, Ramroop S. Spatial distribution and analysis of risk factors associated with Anemia among women of reproductive age: case of 2014 Rwanda demographic and health survey data. The Open Public Health Journal. 2018;11(1).
35. Kibret KT, Chojenta C, D'Arcy E, Loxton D. Spatial distribution and determinant factors of anaemia among women of reproductive age in Ethiopia: a multilevel and spatial analysis. BMJ open. 2019;9(4):e027276.
36. Nankinga O, Aguta D. Determinants of Anemia among women in Uganda: further analysis of the Uganda demographic and health surveys. BMC Public Health. 2019;19(1):1-9.
37. Soofi S, Khan GN, Sadiq K, Ariff S, Habib A, Kureishy S, et al. Prevalence and possible factors associated with anaemia, and vitamin B 12 and folate deficiencies in women of reproductive age in Pakistan: analysis of national-level secondary survey data. BMJ open. 2017;7(12):e018007.
38. Arpey NC, Gaglioti AH, Rosenbaum ME. How socioeconomic status affects patient perceptions of health care: a qualitative study. Journal of primary care & community health. 2017;8(3):169-75.
39. Teshale AB, Tesema GA, Worku MG, Yeshaw Y, Tessema ZT. Anemia and its associated factors among women of reproductive age in eastern Africa: A multilevel mixed-effects generalized linear model. Plos one. 2020;15(9):e0238957.
40. Zimmermann MB, Hurrell RF. Nutritional iron deficiency. The lancet. 2007;370(9586):511-20.

SUPPLEMENTAL TABLE

Table 2. Prevalence of Anemia in the 19 domains in CDHS 2005, CDHS 2010 and 2014

Province	CDHS 2005 (n=486)	CDHS 2010 (n=467)	CDHS 2014 (n=615)
	% of anemia	% of anemia	% of anemia
Banteay Meanchey	62.0	37.0	30.0
Kampong Cham/Tbong Khmum	56.0	50.0	44.0
Kampong Chhnang	53.0	67.0	63.0
Kampong Speu	61.0	48.0	56.0
Kampong Thom	62.0	57.0	62.0
Kandal	53.0	49.0	50.0
Kratie	53.0	78.0	66.0
Phnom Penh	34.0	31.0	46.0
Prey Veng	50.0	60.0	69.0
Pursat	56.0	41.0	52.0
Siem Reap	64.0	56.0	53.0
Svay Rieng	45.0	61.0	64.0
Takeo	64.0	40.0	26.0
Otdar Meanchey	78.0	30.0	59.0
Battambang/Pailin	55.0	58.0	50.0
Kampot/Kep	62.0	76.0	61.0
Preah Sihanouk/Koh Kong	76.0	48.0	65.0

Preah Vihear/Stung Treng	73.0	61.0	59.0
Mondul Kiri/Ratanak Kiri	56.0	49.0	42.0

SUPPLEMENTAL FIGURES

Fig 6. Predicted probability of anemia among pregnant women aged 15-49 years old (n=1,567). Visualized using "ggplot2" package in R.

Fig 7. Predicted probability of anemia among pregnant women aged 15-49 years old (n=1,567). Visualized using "ggplot2" package in R.

Fig 8. Predicted probability of anemia among pregnant women aged 15-49 years old (n=1,567). Visualized using "ggplot2" package in R.

Fig 9. Predicted probability of anemia among pregnant women aged 15-49 years old (n=1,567). Visualized using "ggplot2" package in R.

Figure Click here to access/download;Figure;Fig 1.tiff

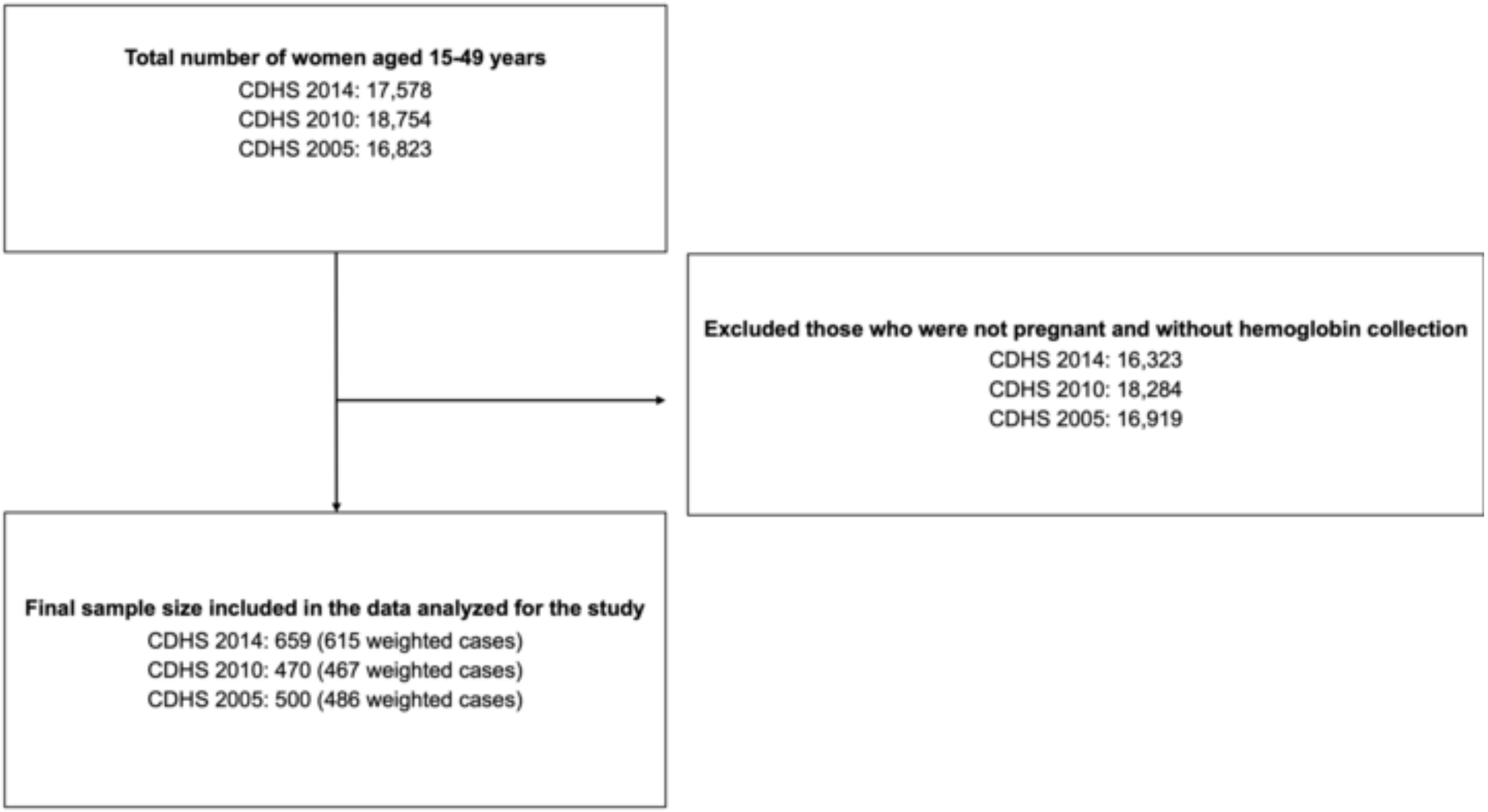

Click here to access/download;Figure;Fig 2.tif

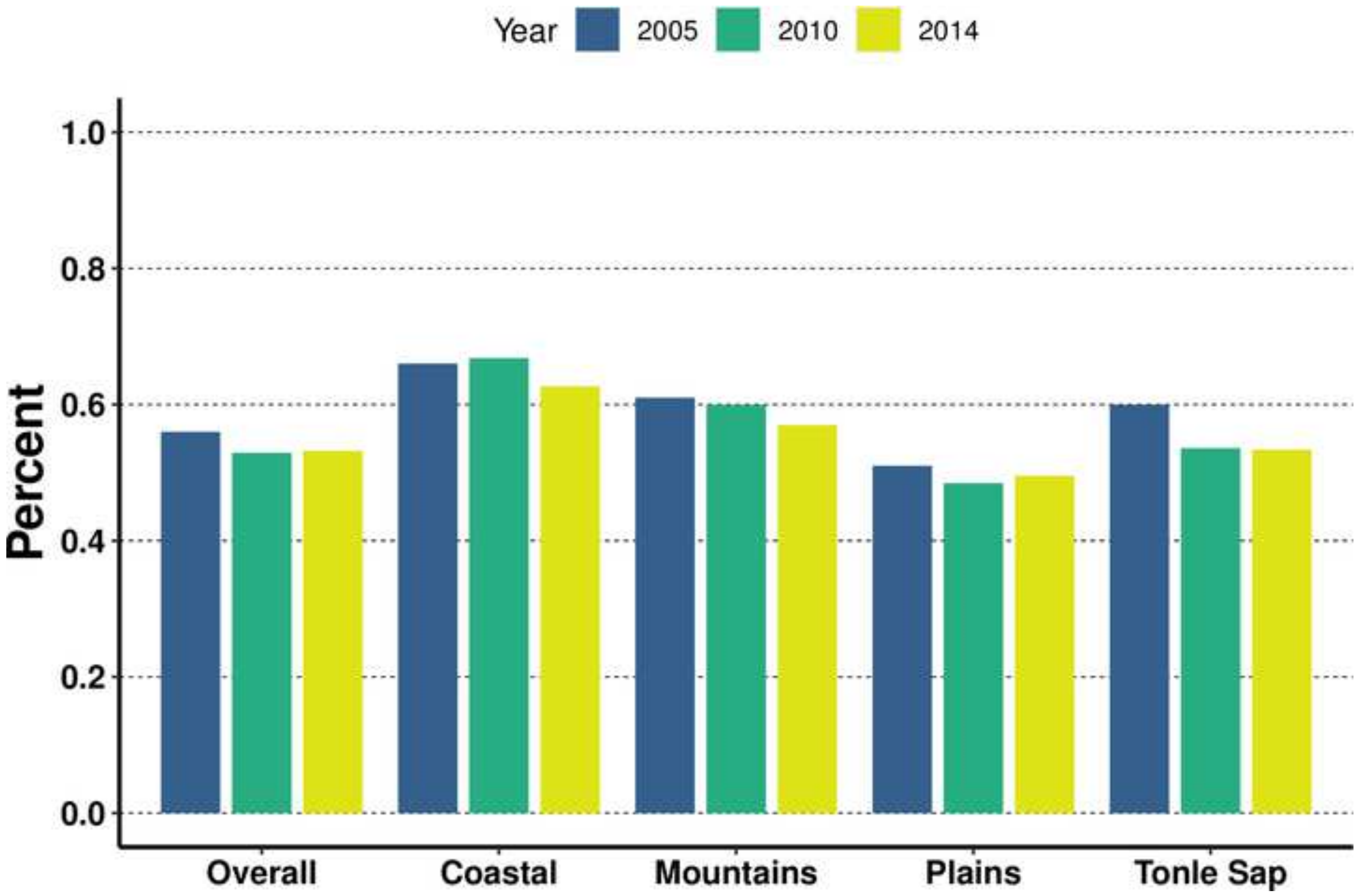

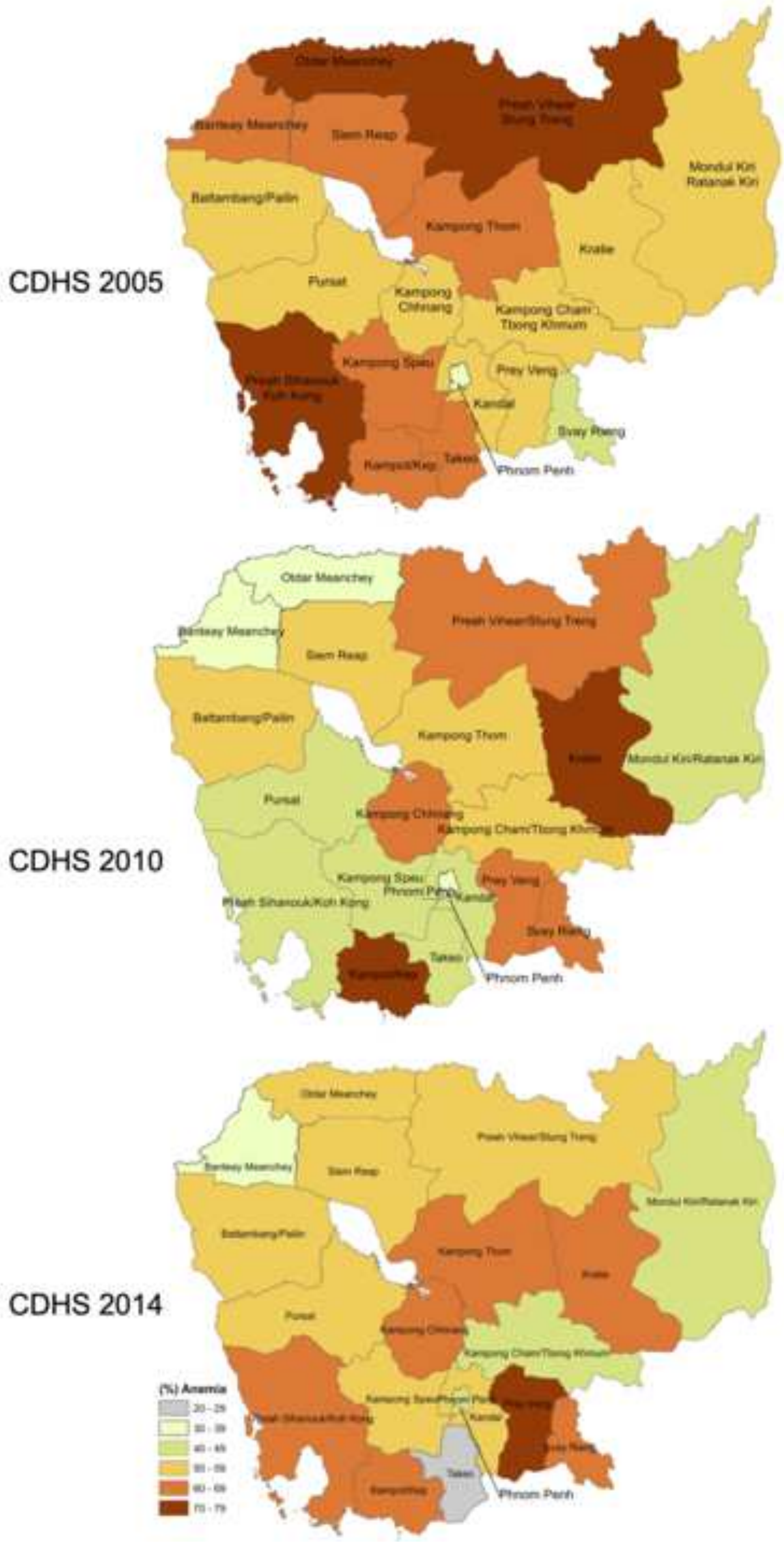
CDHS 2005
Oddar Meanchey
Banteay Meanchey
Siem Reap
Preah Vihear Stung Treng
Mondul Kiri Ratanak Kiri
Battambang/Pailin
Kampong Thom
Kratie
Pursat
Kampong Chhnang
Kampong Cham Tbong Khmum
Kampong Speu
Preah Sihanouk Koh Kong
Prey Veng
Kandal
Svay Rieng
Kampot/Kep
Takeo
Phnom Penh
CDHS 2010
Oddar Meanchey
Banteay Meanchey
Preah Vihear/Stung Treng
Siem Reap
Battambang/Pailin
Kampong Thom
Kratie
Mondul Kiri/Ratanak Kiri
Pursat
Kampong Chhnang
Kampong Speu
Prey Veng
Svay Rieng
Takeo
Phnom Penh
CDHS 2014
Phnom Penh
(%) Anemia

Figure
Click here to access/download;Figure;Fig 4.tif

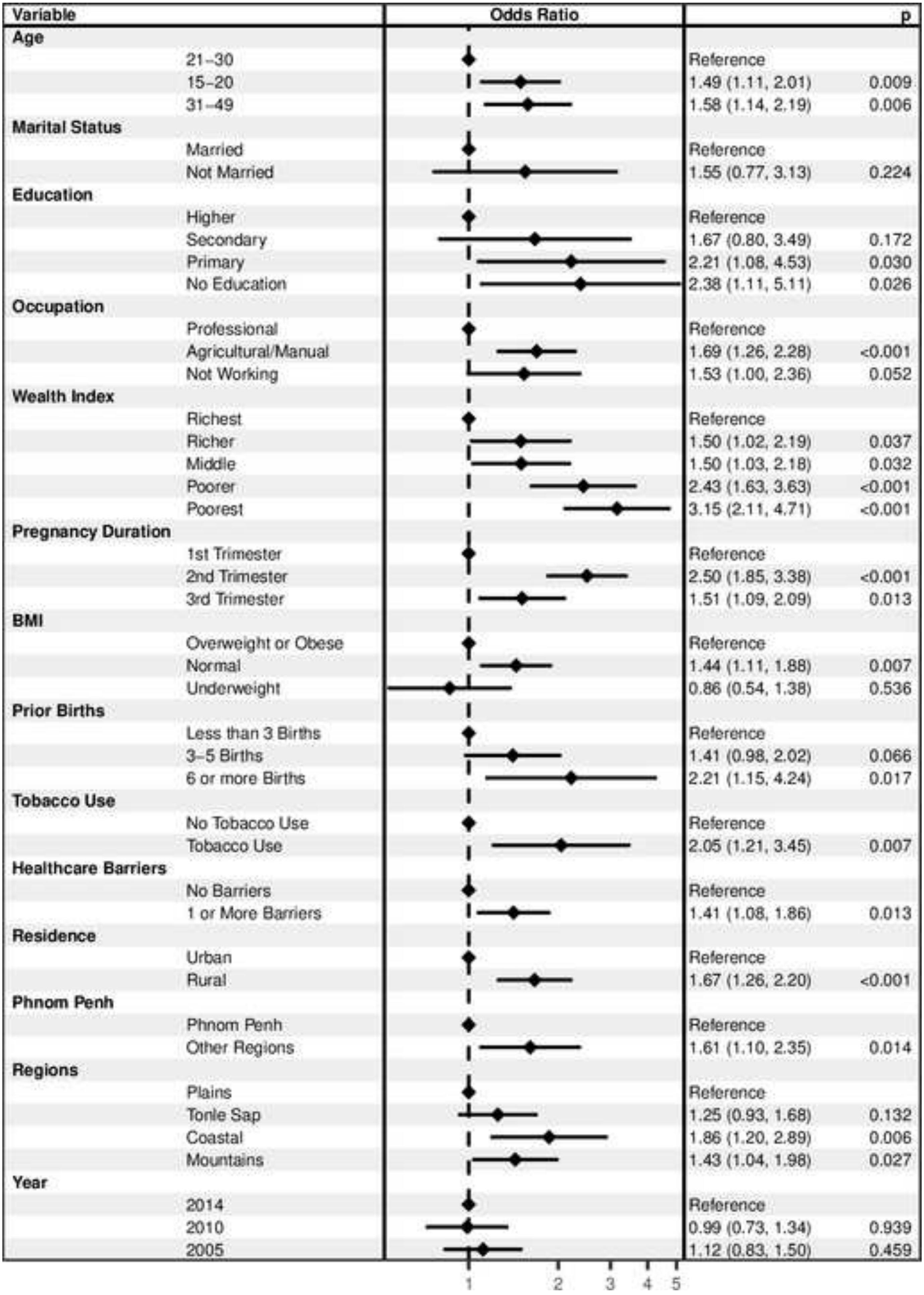

Figure

Click here to access/download;Figure;Fig 5.tif

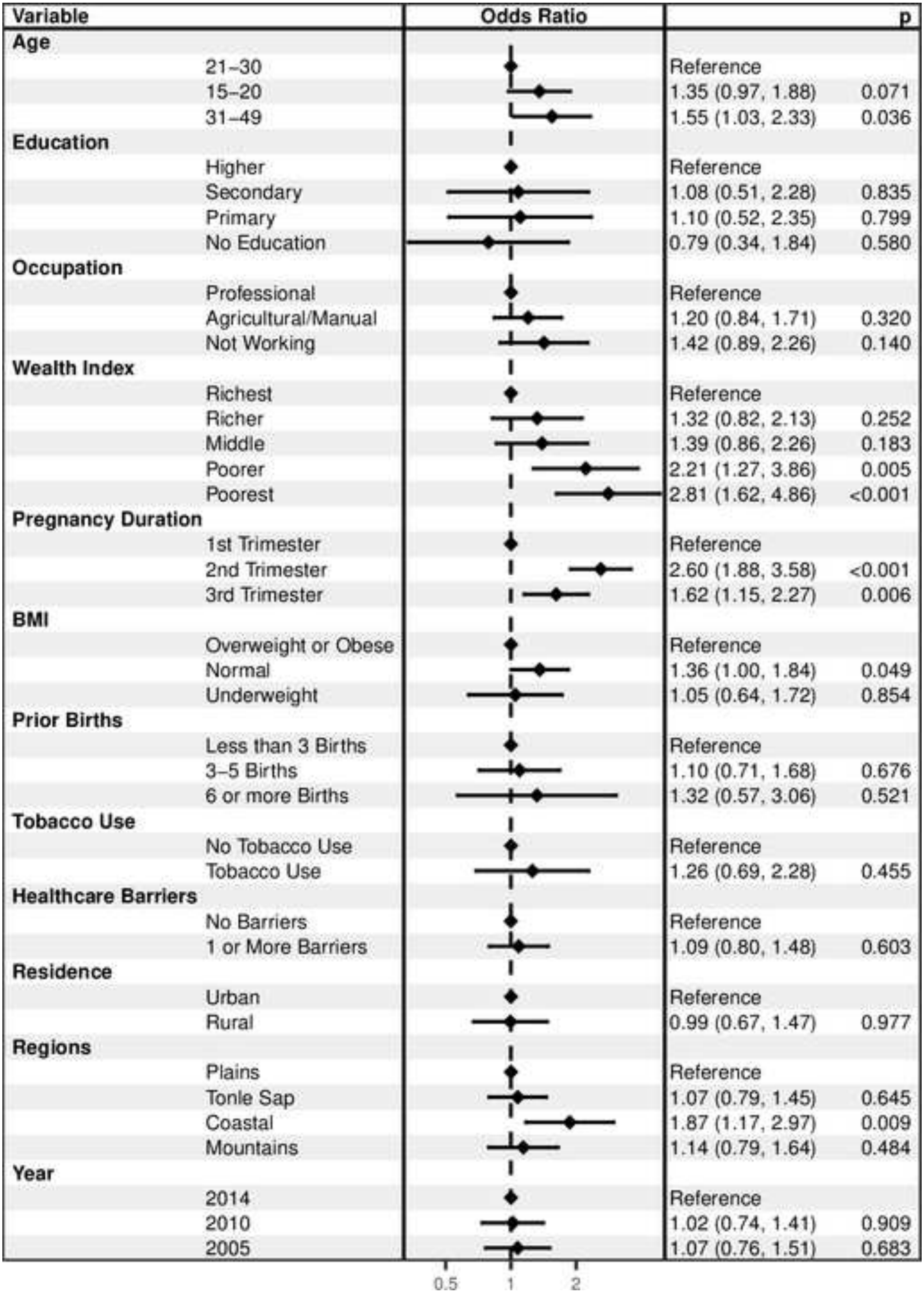

 Click here to access/download;Figure;Fig 6.tif

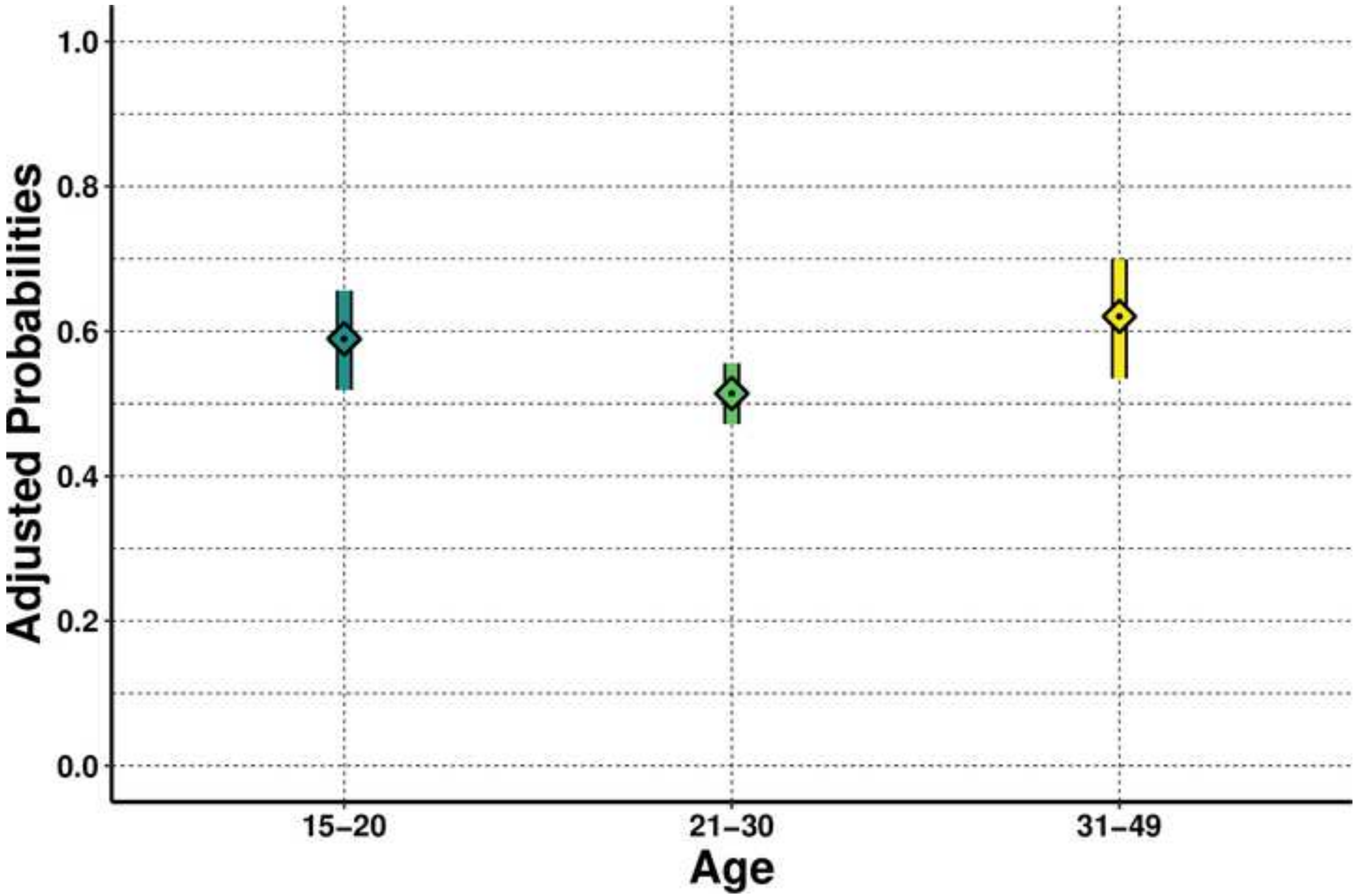

Figure

Click here to access/download;Figure;Fig 7.tif

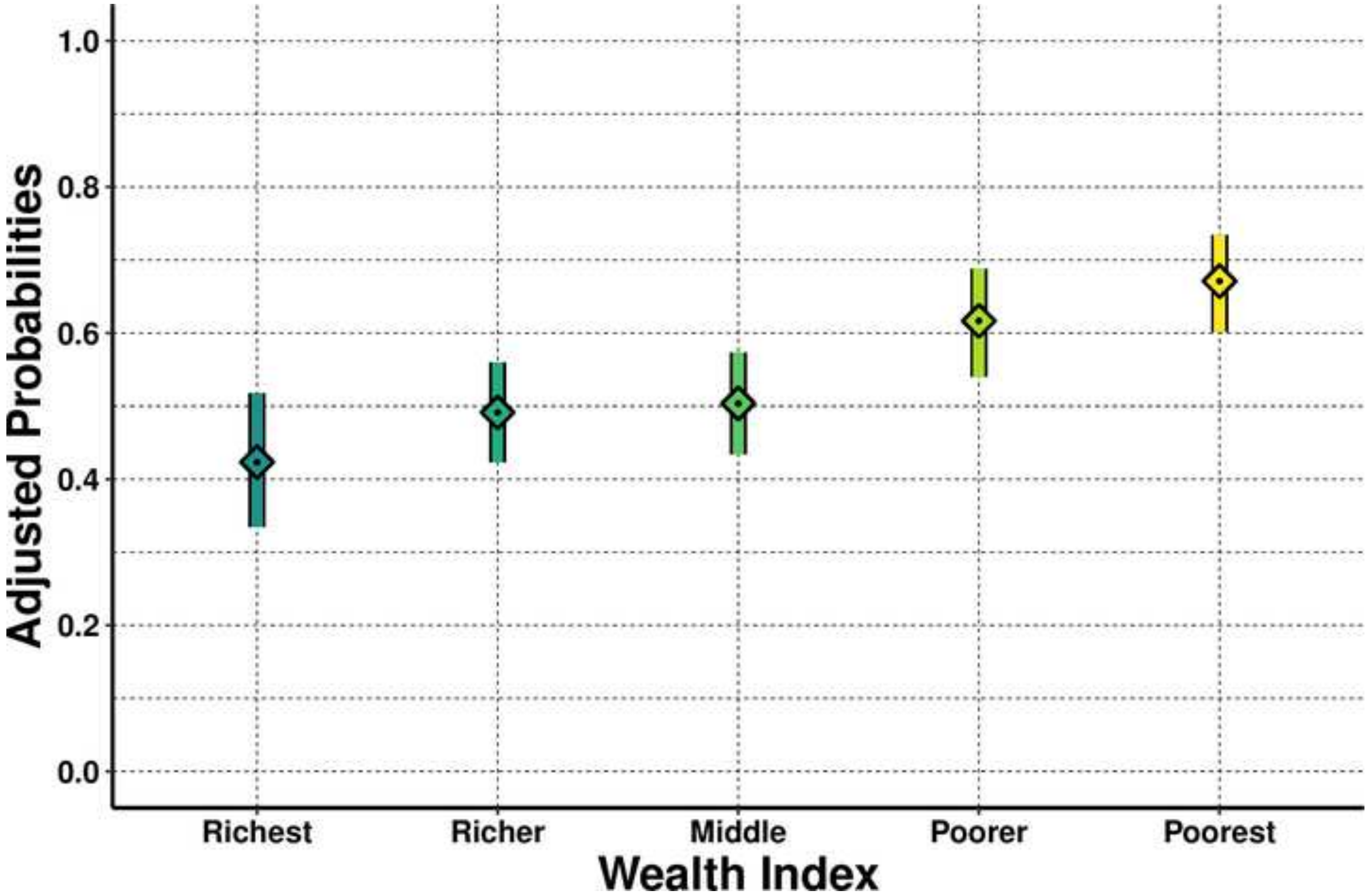

Click here to access/download;Figure;Fig 8.tif

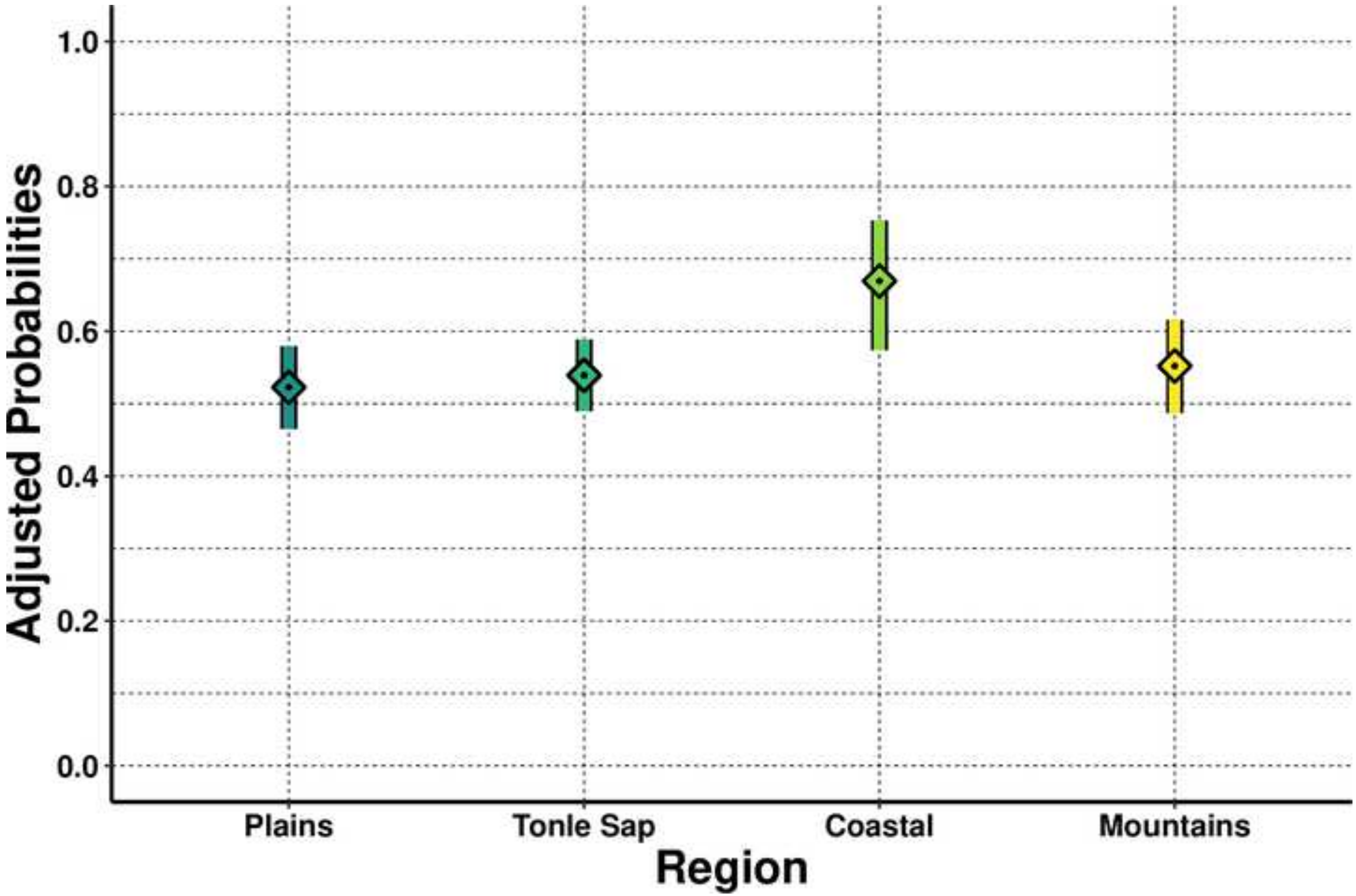

Figure Click here to access/download;Figure;Fig 9.tif

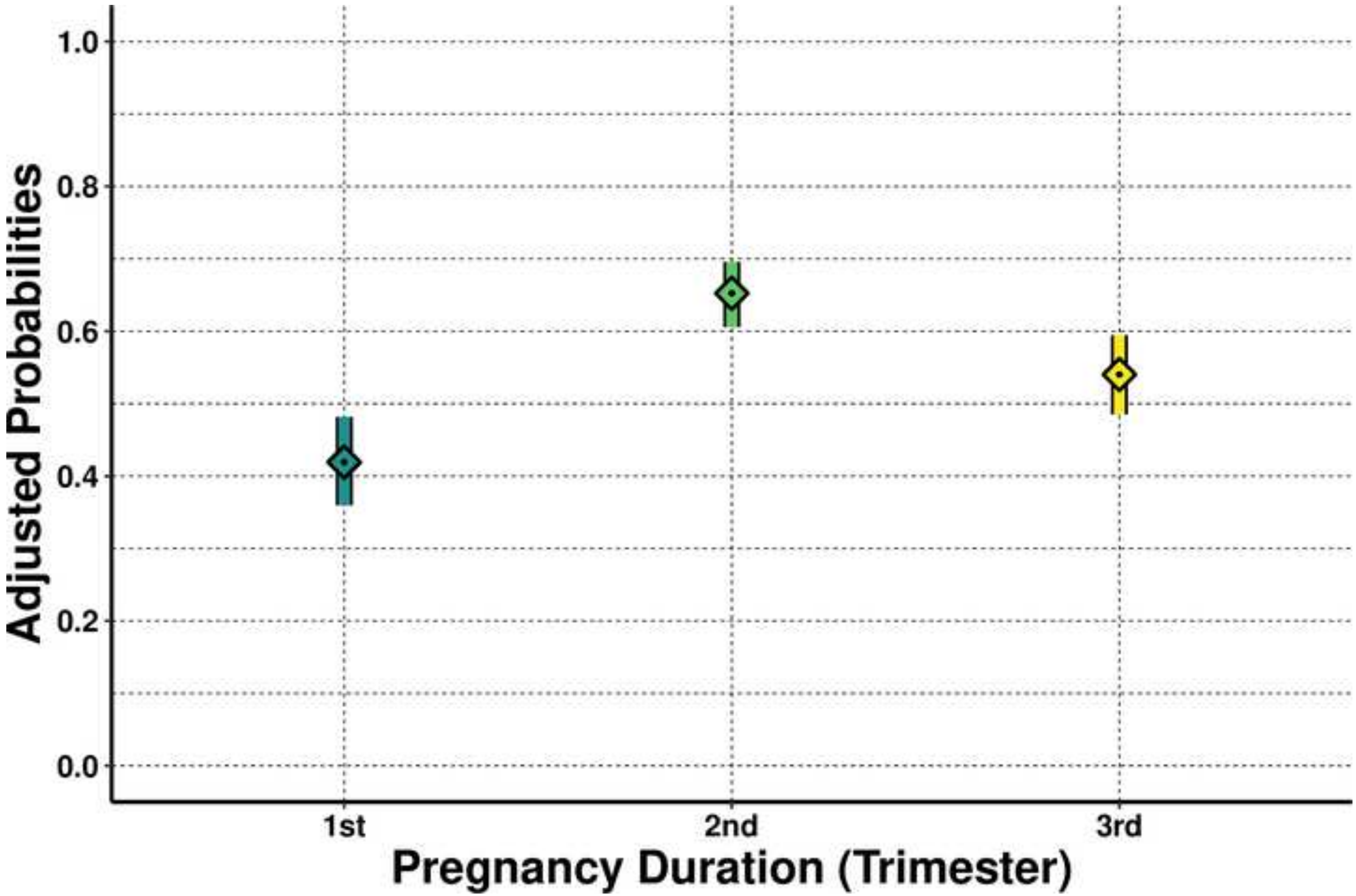

Supplement: Attachment — Submitted filename: PONE-D-22-24121_reviewer.pdf [file pone.0274925.s004.pdf]
