# Supplementary material for: Anemia among pregnant women in Cambodia: A descriptive analysis of temporal and geospatial trends and logistic regression-based examination of factors associated with anemia in pregnant women
Source: PLoS One. 2023 Dec 7;18(12):e0274925. doi: 10.1371/journal.pone.0274925 (PMC10703242; doi:10.1371/journal.pone.0274925)
Supplement: Attachment — Submitted filename: Review-comments.docx [file pone.0274925.s003.docx]

REVIEWER (Dr. D. Ntirampeba) REPORT OF THE MANUSCRIPT TITLED: TRENDS AND FACTORS ASSOCIATED WITH ANEMIA AMONG PREGNANT WOMEN AGED 15-49 YEARS OLD IN CAMBODIA: DATA ANALYSIS OF THE CAMBODIA DEMOGRAPHIC AND HEALTH SURVEYS

1. Recommendation: Major Revision

2. Is the manuscript technically sound, and do the data support the conclusions? Partly

3. Has the statistical analysis been performed appropriately and rigorously? No

4. Does the manuscript adhere to the PLOS Data Policy? Yes

5. Is the manuscript presented in an intelligible fashion and written in standard English? Yes (though few typos are observed)

6. Review Comments to the Author:

Statistical analysis

The authors used logistic models to determine factors associated with anemia among pregnant women aged 15-49 years old in Cambodia. They went on indicating that they fitted bivariate and multivariate logistic regression models. This is confusing as only one response variable was considered. The concepts of bivariate and multivariate are commonly used when the objective of modelling is to jointly analyse two or outcome variables. Thus, I suggest they change to simple and multiple logistic regression models instead of bivariate and multivariate logistic regression models, respectively.

The analysis aimed at describing the temporal and geographic trends of anemia in pregnant. In this paper, the authors have just provided a description of the temporal and geographic trends by merely using a chart and maps of prevalence of anemia. It is not evident that these spatio-temporal trends described in this paper are significant or not. I would suggest they consider using spatio-temporal modelling approach to this end. Furthermore, they need to extend the time window as three data points (3 years) are too little for a temporal trend analysis.

Finally, the analysis is lacking the model diagnostic that ensures that final model is a good fit. This section may include some of the following: Deviance, R-squared for logistic regression, Likelihood ratio-test, Omnibus Test of model coefficients, Hosmer-Lemshow Goodness of fit test, Classification tables & ORC, Wald test, Analysis of residuals, and Validation of Results (Use the validation sample (test) to assess the external validity and practical significance of the model).

The conclusions drawn can be improved once the above issued are considered. This manuscript is presented in an intelligible style and is written in standard English though there are few typos as indicated n the main document.

In addition to availability of the data, the authors should avail the codes (R) used.

7. Would you like your identity revealed to the authors of this submission? No

8. Do you have any potentially competing interests? "None."

9. Do you want to get recognition for this review on Publons (https://publons.com/benefits/reviewers/how)? If you opt in, your Publons profile will automatically be updated to show a verified record of this review in full compliance with the journal's review policy. If you don't have a Publons profile, you will need to first create a free account. Yes

Review comments to authors

Statistical analysis

The authors used logistic models to determine factors associated with anemia among pregnant women aged 15-49 years old in Cambodia. They went on indicating that they fitted bivariate and multivariate logistic regression models. This is confusing as only one response variable was considered. The concepts of bivariate and multivariate are commonly used when the objective of modelling is to jointly analyse two or outcome variables. Thus, I suggest they change to simple and multiple logistic regression models instead of bivariate and multivariate logistic regression models, respectively.

The analysis aimed at describing the temporal and geographic trends of anemia in pregnant. In this paper, the authors have just provided a description of the temporal and geographic trends by merely using a chart and maps of prevalence of anemia. It is not evident that these spatio-temporal trends described in this paper are significant or not. I would suggest they consider using spatio-temporal modelling approach to this end. Furthermore, they need to extend the time window as three data points (3 years) are too little for a temporal trend analysis.

Finally, the analysis is lacking the model diagnostic that ensures that final model is a good fit. This section may include some of the following: Deviance, R-squared for logistic regression, Likelihood ratio-test, Omnibus Test of model coefficients, Hosmer-Lemshow Goodness of fit test, Classification tables & ORC, Wald test, Analysis of residuals, and Validation of Results (Use the validation sample (test) to assess the external validity and practical significance of the model).

The conclusions drawn can be improved once the above issued are considered. This manuscript is presented in an intelligible style and is written in standard English though there are few typos as indicated n the main document.

In addition to availability of the data, the authors should avail the codes (R) used.

Reviewer

Dr D. Ntirampeba
