# Supplementary material for: Anemia among pregnant women in Cambodia: A descriptive analysis of temporal and geospatial trends and logistic regression-based examination of factors associated with anemia in pregnant women
Source: PLoS One. 2023 Dec 7;18(12):e0274925. doi: 10.1371/journal.pone.0274925 (PMC10703242; doi:10.1371/journal.pone.0274925)
Supplement: Attachment — Submitted filename: Response to Reviewers.docx [file pone.0274925.s005.docx]

PONE-D-22-24121

**Trends and factors associated with anemia among pregnant women aged 15-49 years old in Cambodia: Data analysis of the Cambodia Demographic and Health Surveys**

Journal Requirements:

When submitting your revision, we need you to address these additional requirements.

1. Please ensure that your manuscript meets PLOS ONE's style requirements, including those for file naming. The PLOS ONE style templates can be found at

<https://journals.plos.org/plosone/s/file?id=wjVg/PLOSOne_formatting_sample_main_body.pdf>

and

<https://journals.plos.org/plosone/s/file?id=ba62/PLOSOne_formatting_sample_title_authors_affiliations.pdf>

a. **We believe that we have followed these style requirements. We have used the PACE tool to revise any figures as needed. We do not want issues with formatting to constitute a barrier to publication, so if we have missed something, please let us know, we are more than willing to address any formatting errors that we may have missed.**

2. We note that Figure 3 in your submission contain [map/satellite] images which may be copyrighted. All PLOS content is published under the Creative Commons Attribution License (CC BY 4.0), which means that the manuscript, images, and Supporting Information files will be freely available online, and any third party is permitted to access, download, copy, distribute, and use these materials in any way, even commercially, with proper attribution. For these reasons, we cannot publish previously copyrighted maps or satellite images created using proprietary data, such as Google software (Google Maps, Street View, and Earth). For more information, see our copyright guidelines:

<http://journals.plos.org/plosone/s/licenses-and-copyright>.

a. **We have noted in the manuscript that these maps were created by our own team based on data from the Cambodia DHS and using publicly available shapefiles for Cambodia from the DHS shapefile repository.**

Reviewers' comments:

Reviewer's Responses to Questions

**Comments to the Author**

1. Is the manuscript technically sound, and do the data support the conclusions?

The manuscript must describe a technically sound piece of scientific research with data that supports the conclusions. Experiments must have been conducted rigorously, with appropriate controls, replication, and sample sizes. The conclusions must be drawn appropriately based on the data presented.

Reviewer #1: Partly

Reviewer #2: Partly

- **Revised according to recommendations provided by the two anonymous reviewers**

2. Has the statistical analysis been performed appropriately and rigorously?

Reviewer #1: Yes

Reviewer #2: No

- **Revised according to recommendations provided by the two anonymous reviewers**

3. Have the authors made all data underlying the findings in their manuscript fully available?

The [PLOS Data policy](http://www.plosone.org/static/policies.action#sharing) requires authors to make all data underlying the findings described in their manuscript fully available without restriction, with rare exception (please refer to the Data Availability Statement in the manuscript PDF file). The data should be provided as part of the manuscript or its supporting information, or deposited to a public repository. For example, in addition to summary statistics, the data points behind means, medians and variance measures should be available. If there are restrictions on publicly sharing data—e.g. participant privacy or use of data from a third party—those must be specified.

Reviewer #1: No

Reviewer #2: Yes

- **These are secondary data that are publicly available by request from the DHS**

4. Is the manuscript presented in an intelligible fashion and written in standard English?

PLOS ONE does not copyedit accepted manuscripts, so the language in submitted articles must be clear, correct, and unambiguous. Any typographical or grammatical errors should be corrected at revision, so please note any specific errors here.

Reviewer #1: Yes

Reviewer #2: Yes

5. Review Comments to the Author

Please use the space provided to explain your answers to the questions above. You may also include additional comments for the author, including concerns about dual publication, research ethics, or publication ethics. (Please upload your review as an attachment if it exceeds 20,000 characters)

**Reviewer #1:**

Trends and factors associated with anemia among pregnant women aged 15-49 years old in Cambodia: Data analysis of the Cambodia Demographic and Health Surveys
Using three rounds of the Cambodian DHS dataset, authors aimed to evaluate the pattern and contributing factors of anemia among pregnant women. Overall, the manuscript is nicely written, incorporates use of the proper statistical analysis, and draws a conclusion based on the results. Nevertheless, there are a number of issues with this version that must be addressed before it can move further.

Including line number could be helpful to locate the comments

a. **We are grateful to reviewer 1 for their thoughtful and helpful feedback. We have endeavored to revise our manuscript in line with your recommendations. In instances where we have opted to use an alternative approach, we have explained why. As requested, we have included line numbers in our revised manuscript to ease the review process.**

Title

2. Please revise the title with “**Trends and factors associated with anemia among pregnant women in Cambodia: analysis of 2005 to 2014 Cambodia Demographic and Health Surveys**”
   a. **We have revised as suggested**

Abstract

3. Introduction: Remove the word “roughly 37% (32 million)” and replace with suitable word here.
   a. **We have revised as suggested**
4. "Women in developing countries are at higher risk of anemia due to micronutrient deficiencies, hemoglobinopathies, infections, or other socio-demographic factors, especially among pregnant women" this sentence is not justifiable because anemia is highly prevalent among both under five children and women of reproductive age. So, please revise this sentence.
   a. **We have revised**
5. Replace describes with “aimed”
   a. **We have revised as suggested**

Results

6. Please also mention the changing pattern of anemia geographically.
   a. **We have revised as suggested**

Manuscript
Introduction:

7. Replace g/dl with “g/dL”
   a. **We have revised as suggested**
8. "In 2019, it was estimated that anemia affected approximately 37% (32 million) of pregnant women worldwide…….." you can catch this information
   a. **We have revised as suggested**
9. Reference 4 do not have the mention information. Please insert the suitable sources here. You can get this information in above mentioned link.

a. **We have revised**

10. Is reference 9 reliable source?
    a. **We have revised**
11. Given that other research has already been published utilizing the CDHS dataset, the authors should discuss other studies that used the same data and explain what makes their study unique. Most likely demonstrating trend analysis, geographic variation, and the significance of each. Furthermore, the study did not break out the variables affecting anemia by geographical area. The report only lists the geographic regions where anemia is most common. Given that both studies use the same data, the author needs to explain why this one is more significant than the others indicated above.
    a. **We have revised to better convey the literature that has already been published and how our study offers a unique contribution. Please note that an informal research abstract was previously “published” in association with a research conference in Cambodia that represents an earlier iteration of this research, which has been revised substantially since. We have referenced this directly now in the text, but would like to indicate that this is not a competing research study. To our knowledge, only one other study has partial overlap with ours, which was regionally focused, was not focused on pregnant women, and did not cover the same degree of detail specific to Cambodia that we are covering in our article.**

Materials and methods

12. Fig 1 do not provide complete information regarding the sample size selection process. How did you select the (sub) sample? What are the inclusion/exclusion criteria? Detail information is required to make clearer.
    a. **We have revised the figure to delineate the exclusion criteria more clearly.**
13. It would be preferable if you included all of these coding strategies in a table and uploaded it as a supporting document. What matters most in the manuscript is how you recategorized the predictor variables.
    a. **We have described the coding scheme of all of our variables comprehensively within the text and indicated that our analytic code is available upon request.**
14. Not multivariate, use “multivariable logistic regression” throughout the manuscript.
    a. **We have considered the recommendations of both reviewers, reviewed the literature and online discussions related to naming conventions for these terms, and consulted with colleagues external to this study. We have opted to revise the use of bivariate/multivariate to unadjusted/adjusted.**
15. Authors have mentioned "A significant level of any covariates at p-value < 0.15 were included in the multivariate logistic regression analysis" why did you include only <0.15 value? Most of the papers have mentioned <0.2.
    a. **We have revised to highlight that our primary inclusion criterion is theoretical relevance. The additional criteria of <0.15 is used as we favor a more conservative level for the inclusion criterion.**
16. The authors reported on the multicollinearity check, but they failed to indicate the cutoff value that was employed in this study.
    a. **We have revised the manuscript to explain that concerns with correlation among independent variables were assessed using a combination of cross tabulation chi-square tests and Cramer’s v scores; all results suggested weak to moderately weak correlations. Further consideration of whether to include a given variable was based on broader evaluations of model fit.**

Results

17. Instead of pooled data, Table 1 should display data from all three DHS survey rounds. It is logical. Therefore, please revise it accordingly.
    a. **As observed by reviewer 2, our results suggest minimal variation in anemia over time and these variations are not statistically significant.**

**Moreover, all of our interaction terms evaluating effect modification of the independent variables included in our analysis by survey year were not statistically significant, indicating that there were no statistically significant variations over time in the associations between these independent variables and anemia. Given this lack of variation, we have opted for an elegant, parsimonious representation of the overall distributions of characteristics in our study population.**

**We believe this is the ideal format for presenting this descriptive information to highlight the important takeaways from our study. However, if the manuscript is deemed unpublishable unless we abide by the recommendation to revise Table 1 and display the data disaggregated by survey year, we will comply.**

Discussion

18. The authors have mentioned that "The elevated prevalence of anemia among pregnant women in Cambodia may be explained by dietary habits, deficiencies in micronutrients such as iron or vitamin A, hemoglobinopathy, malaria infections, and helminths" that means does dietary pattern is different from the neighboring countries? Please explain it in detail.
    a. **We noted these factors in the manuscript as potential pathways through which the elevated prevalence of anemia may result. We have revised the manuscript to further note that this requires further evaluation. We appreciate the encouragement to describe this in detail; however, explicating potential differences in dietary patterns across neighboring countries is beyond the scope of this manuscript and would better fit in a follow-up study with detailed nutritional data from multiple countries in Southeast Asia.**
19. The mentioned programs are the public health intervention implemented in Cambodia. It would be better if you mention the compliance and effectiveness of such program to mitigate the anemia among pregnant women in Cambodia.
    a. **We have revised the manuscript to include additional information on compliance with these types of interventions.**
20. Age is still contradictory. Some studies concluded that getting older is protective, while others countered that it makes women more susceptible to anemia. Please discuss.

Some studies are:

<https://bmjopen.bmj.com/content/11/3/e041982.long#ref-39>
<https://bmjopen.bmj.com/content/bmjopen/9/Suppl_3/1.full.pdf>

<https://pubmed.ncbi.nlm.nih.gov/23516270/>

a. **We reviewed the suggested studies; from our reading, these examples are less relevant as they represent broader associations among women of reproductive age—the articles that we have referenced are for studies specifically focused on pregnant women, these studies consistently note an increased risk for pregnant women at older ages.**

21. Why pregnant women having normal BMI had higher odds of anemia compared to overweight/obese women?
    a. **As noted in the manuscript, this association requires further evaluation with additional data.**

22. Disparities of anemia according to geographical pattern might be explained here:

<https://bmjopen.bmj.com/content/11/3/e041982.long#ref-64>

a. **Included**

**Reviewer #2:**

Statistical analysis

**Our thanks to reviewer 2 for their thoughtful and helpful feedback. We have endeavored to revise our manuscript in line with your recommendations.**

1. The authors used logistic models to determine factors associated with anemia among pregnant women aged 15-49 years old in Cambodia. They went on indicating that they fitted bivariate and multivariate logistic regression models. This is confusing as only one response variable was considered. The concepts of bivariate and multivariate are commonly used when the objective of modelling is to jointly analyse two or outcome variables. Thus, I suggest they change to simple and multiple logistic regression models instead of bivariate and multivariate logistic regression models, respectively.
   a. **We have considered the recommendations of both reviewers, reviewed the literature and online discussions related to naming conventions for these terms noting that there is unfortunate ambiguity with how these terms have been used across various disciplines, and consulted with colleagues external to this study. We have opted to revise the use of bivariate/multivariate to unadjusted/adjusted throughout the manuscript.**
2. The analysis aimed at describing the temporal and geographic trends of anemia in pregnant. In this paper, the authors have just provided a description of the temporal and geographic trends by merely using a chart and maps of prevalence of anemia. It is not evident that these spatio-temporal trends described in this paper are significant or not. I would suggest they consider using spatio-temporal modelling approach to this end. Furthermore, they need to extend the time window as three data points (3 years) are too little for a temporal trend analysis.
   a. **We have revised the manuscript, both in the abstract and in the main text, to indicate that this analysis has two primary aims. The first aim is to *describe* temporal and geographic trends of anemia in pregnant women as noted by the reviewer. The second is to identify factors associated with anemia among pregnant women.**
1. **Aim 1: The reviewer is correct that our results suggest minimal variation over time and that these variations are not statistically significant. While the differences across the temporal trend of anemia is not statistically significant, we believe that these results are substantively important — we believe it is important to document evidence that suggests little improvement in anemia levels among pregnant women during the time considered.**

**We concur with the reviewer that 3 points in time is less than ideal and have noted this as a limitation in our discussion. We likewise agree with the reviewer’s recommendations for a more sophisticated analytic approach and will look forward to engaging in that analysis once additional data become available. However, we believe that the current results have scientific importance worthy of documentation at the present time.**

2. **Aim 2: After describing the temporal and spatial trends in anemia, albeit with the limitations that we have acknowledged, we focus most of our analysis on examining factors associated with pregnant women in Cambodia. For this analysis, we did evaluate potential effect modification for all considered factors through the modeling of interaction terms with the temporal variable for year; however, the results from these analyses were not statistically significant. Given the lack of statistical significance of temporal difference, we were not surprised by the lack of effect modification with other factors. We have endeavored to revise the manuscript to delineate these observations more clearly in the discussion.**
3. Finally, the analysis is lacking the model diagnostic that ensures that final model is a good fit. This section may include some of the following: Deviance, R-squared for logistic regression, Likelihood ratio-test, Omnibus Test of model coefficients, Hosmer-Lemshow Goodness of fit test, Classification tables & ORC, Wald test, Analysis of residuals, and Validation of Results (Use the validation sample (test) to assess the external validity and practical significance of the model).
   The conclusions drawn can be improved once the above issued are considered.
   a. **We appreciate these recommendations—tests of model fit were performed previously, but not reported. We now note that Pseudo-R2, Deviance, AIC, and BIC values were used to evaluate model fit and that evaluation of these indicators support the final, fully adjusted model.**
4. This manuscript is presented in an intelligible style and is written in standard English though there are few typos as indicated in the main document.
   a. **We have revised to address any typos that we were able to identify – if there are specific instances that we missed that the reviewers would like to direct to our attention, we would be most grateful.**
5. In addition to availability of the data, the authors should avail the codes (R) used.
   a. **The analysis is based on secondary data that are publicly available by request from the DHS. We requested and received authorization to use these data from the DHS for analytic purposes – we do not have authorization to take on the role of a data disseminator of their data.**
   b. **We have indicated within the text that our analytic code is available upon request.**

**PLOS ONE: Edits requested on your submission PONE-D-22-24121R1 - [EMID:efcf5f5c135b2259]**

**PONE-D-22-24121R1**

**Trends and factors associated with anemia among pregnant women in Cambodia: Analysis of 2005 to 2014 Cambodia Demographic and Health Surveys**

**Mr. Samnang Um**

**Dear Dr. Um,**

**We've checked your submission and before we can proceed, we need you to address the following issues:**

**1. Thank you for your response to our copyright inquiry, which reads as follows: "We have noted in the manuscript that these maps were created by our own team based on data from the Cambodia DHS and using publicly available shapefiles for Cambodia from the DHS shapefile repository."**

**Regarding the potential copyright of Figure 3, please respond to the below prompts:**

**A. For the above figure, please provide relevant links to the publicly available shapefiles used. Please provide any relevant links to any relevant copyright information.**

**Shapefiles of Cambodia provinces were obtained from the publicly available Spatial Data Repository associated with the DHS website at [https://spatialdata.dhsprogram.com/covariates/].**

**B. For the above figure, please provide relevant links to the data utilized in the creation of this figure. Please also provide any relevant links to any relevant copyright information.**

**Publicly datasets available on the Demographic and Health Surveys program website at [https://dhsprogram.com/data/available-datasets.cfm].**

**C. Was any software (e.g. ArcGIS) used in the creation of this figure?**

**A temporal series of maps illustrating provincial variation in the prevalence of anemia was also prepared by our team using ArcGIS software version 10.8.**

**Citation: Desktop A. Release 10.8. 1.(2020). ESRI (Environmental Systems Research Institute), Redlands, CA.**

**We look forward to your response.**

**We've returned your manuscript to your account. Please resolve these issues and resubmit your manuscript within 21 days. If you need more time, please email the journal office at plosone@plos.org. We are happy to grant extensions of up to one month past this due date. If we do not hear from you within 21 days, we will withdraw your manuscript.**

**Please log on to PLOS Editorial Manager at https://www.editorialmanager.com/pone/ to access your manuscript. You will find your manuscript in the 'Submissions Sent Back to Author' link under the New Submissions menu. Be sure to remove your previous manuscript file if you are uploading a new file in response to these requests. After you've made the changes requested above, please be sure to view and approve the revised PDF after rebuilding the PDF to complete the resubmission process.**

**We are requesting these changes to comply with the PLOS ONE submission guidelines (https://journals.plos.org/plosone/s/submission-guidelines). Please note that we won't send your manuscript for review until you have resolved the above requests.**

**Thank you for submitting your work to PLOS ONE and supporting our mission of Open Science.**

**Kind regards,**

**Rose Ann Joyce Sagun Puetes**

**PLOS ONE**
