# Supplementary material for: Anemia among pregnant women in Cambodia: A descriptive analysis of temporal and geospatial trends and logistic regression-based examination of factors associated with anemia in pregnant women
Source: PLoS One. 2023 Dec 7;18(12):e0274925. doi: 10.1371/journal.pone.0274925 (PMC10703242; doi:10.1371/journal.pone.0274925)
Supplement: Attachment — Submitted filename: Response to Reviewers-R2.docx [file pone.0274925.s006.docx]

**Authors’ point-by-point responses to the editor and reviewers**

PONE-D-22-24121R1

**Trends and factors associated with anemia among pregnant women in Cambodia: Analysis of 2005 to 2014 Cambodia Demographic and Health Surveys**

**Anemia among Pregnant Women in Cambodia: A Descriptive Analysis of Temporal and Geospatial Trends and Logistic Regression-Based Examination of Factors Associated with Anemia in Pregnant Women**

PLOS ONE

**Journal Requirements:**

1. Please review your reference list to ensure that it is complete and correct. If you have cited papers that have been retracted, please include the rationale for doing so in the manuscript text, or remove these references and replace them with relevant current references. Any changes to the reference list should be mentioned in the rebuttal letter that accompanies your revised manuscript. If you need to cite a retracted article, indicate the article’s retracted status in the References list and also include a citation and full reference for the retraction notice.

We believe that we have followed the formatting of the standard PLOS restyle for all references.

**Reviewers' comments:**

Reviewer's Responses to Questions

**Comments to the Author**

1. If the authors have adequately addressed your comments raised in a previous round of review and you feel that this manuscript is now acceptable for publication, you may indicate that here to bypass the “Comments to the Author” section, enter your conflict-of-interest statement in the “Confidential to Editor” section, and submit your "Accept" recommendation.

Reviewer #1: (No Response)

2. Is the manuscript technically sound, and do the data support the conclusions?

The manuscript must describe a technically sound piece of scientific research with data that supports the conclusions. Experiments must have been conducted rigorously, with appropriate controls, replication, and sample sizes. The conclusions must be drawn appropriately based on the data presented.

Reviewer #1: Yes

3. Has the statistical analysis been performed appropriately and rigorously?

Reviewer #1: I Don't Know

4. Have the authors made all data underlying the findings in their manuscript fully available?

The [PLOS Data policy](http://www.plosone.org/static/policies.action#sharing) requires authors to make all data underlying the findings described in their manuscript fully available without restriction, with rare exception (please refer to the Data Availability Statement in the manuscript PDF file). The data should be provided as part of the manuscript or its supporting information, or deposited to a public repository. For example, in addition to summary statistics, the data points behind means, medians and variance measures should be available. If there are restrictions on publicly sharing data—e.g. participant privacy or use of data from a third party—those must be specified.

Reviewer #1: No

5. Is the manuscript presented in an intelligible fashion and written in standard English?

PLOS ONE does not copyedit accepted manuscripts, so the language in submitted articles must be clear, correct, and unambiguous. Any typographical or grammatical errors should be corrected at revision, so please note any specific errors here.

Reviewer #1: Yes

6. Review Comments to the Author

Please use the space provided to explain your answers to the questions above. You may also include additional comments for the author, including concerns about dual publication, research ethics, or publication ethics. (Please upload your review as an attachment if it exceeds 20,000 characters)

Reviewer #1: Manuscript ID: PONE-D-22-24121R1
Title: Trends and factors associated with anemia among pregnant women in Cambodia: Analysis of 2005 to 2014 Cambodia Demographic and Health Surveys

The most of the comments have been addressed by the authors; nevertheless, a few remarkable issues remain that should be addressed before a decision is made.

General comments

1.Also, I couldn't find information about the construction of forest plots for Figs 4 and 5. Please provide information in the manuscript about how forest plots were constructed.

Forest plots were generated using the forest model package in R.

3. Authors have claimed to evaluate temporal and geographical trends in anemia among pregnant women using nationally representative data in Cambodia. However, I haven't noticed the findings of geographical trends in this study. It needs robust spatial analysis.

We have revised

4. To display the descriptive results at different times, I still strongly recommend presenting the descriptive data for all three rounds of surveys in Table 1.

We have revised Table 1 of the manuscript as suggested.

Specific comments
1. The X axis label in Fig 2 needs to be corrected to a percentage, which looks confusing.

We have revised the figure to a percentage as suggested.

2. Discussion: In Cambodia, despite the implementation of various public health programs, the prevalence of anemia among pregnant women remains stagnant. Please discuss the plausible reason and ways to mitigate the problem.

It is not only pregnant women but also not pregnant women. It is related to genetic hemoglobin disorders like Thalassemia. It could not treat with medications. The prevalence rate of Thalassemia is 50 to 60% in Cambodia.

3. Why pregnant women with a normal BMI were more likely to have anemia compared to pregnant women who were overweight or obese? Please explain the plausible physiological mechanism.

It may be related to food. The iron content of food and the food habit of pregnant women in Cambodia are not clear, but it may simply be that the more eating is more intake iron. Need a nutrition survey for pregnant women in Cambodia.

4. I had recommended including the evidence of disparities of anemia according to geographical patterns in the previous round, but I could not locate it.
<https://bmjopen.bmj.com/content/11/3/e041982>

Future research should consider a formal spatial analysis for enhancing the scientific rigor of geospatial trends as recommended paper.
