# Supplementary material for: Anemia among pregnant women in Cambodia: A descriptive analysis of temporal and geospatial trends and logistic regression-based examination of factors associated with anemia in pregnant women
Source: PLoS One. 2023 Dec 7;18(12):e0274925. doi: 10.1371/journal.pone.0274925 (PMC10703242; doi:10.1371/journal.pone.0274925)
Supplement: Attachment — Submitted filename: Response-to-Reviewers-R3.docx [file pone.0274925.s007.docx]

**Authors’ point-by-point responses to the editor and reviewers**

We thank the editor and anonymous reviewers for the opportunity to revise and resubmit our manuscript. All of the reviewers provided us with thoughtful and thorough evaluations of our paper. We believe that the revisions we have made based on these comments have allowed us to develop a much-improved manuscript. We feel that we have addressed all of the reviewer’s comments. However, if we have missed something, we are more than willing to make the necessary changes going forward. Our responses to the specific points raised by each reviewer are outlined in the summary of changes outlined herein.

Additional Editor Comments:

Together with the new requests from the reviewers, these requests from previous reviews have not been addressed in the current version and should be in the next revision.

1.I am not convincing with the using the "unadjusted/adjusted" instead of bivariate/multivariable. As suggested by the reviewer in the previous round, I would rather recommend to use simple and multiple logistic regression models. Also, I couldn't find information about the construction of forest plots for Figs 4 and 5. Please provide information in the manuscript about how forest plots were constructed.

**We have revised the manuscript to use the terminology requested by the reviewer and also included a description within the text indicating the use and means of creating the forest plots.**

The authors provide details on how the Forest plot was created in R in the legend of the figure but this should instead be detailed in the Methods section of the paper.

**We have included a description within the text indicating the use and means of creating the forest plots as requested.**

2. I had asked this query in the previous round "A significant level of any covariates at p-value < 0.15 were included in the multivariate logistic regression analysis" why did you include only <0.15 value? Most of the papers have used <0.2. The author claims the p-value of 0.15 is relevant from a theoretical standpoint. It is not clear. Please clarify it in simple and understandable way.

**We thank the reviewer for their original and additional observations regarding this issue. We agree with the reviewer’s observation in their additional feedback that is listed below regarding this issue of concern. We have revised the manuscript to indicate that justification for inclusion in the model were based on theoretical grounds.**

[Note: HTML markup is below. Please do not edit.]

Reviewers' comments:

Reviewer's Responses to Questions

Comments to the Author

1. If the authors have adequately addressed your comments raised in a previous round of review and you feel that this manuscript is now acceptable for publication, you may indicate that here to bypass the “Comments to the Author” section, enter your conflict of interest statement in the “Confidential to Editor” section, and submit your "Accept" recommendation.

Reviewer #3: (No Response)

2. Is the manuscript technically sound, and do the data support the conclusions?

The manuscript must describe a technically sound piece of scientific research with data that supports the conclusions. Experiments must have been conducted rigorously, with appropriate controls, replication, and sample sizes. The conclusions must be drawn appropriately based on the data presented.

Reviewer #3: Partly

3. Has the statistical analysis been performed appropriately and rigorously?

Reviewer #3: No

4. Have the authors made all data underlying the findings in their manuscript fully available?

The PLOS Data policy requires authors to make all data underlying the findings described in their manuscript fully available without restriction, with rare exception (please refer to the Data Availability Statement in the manuscript PDF file). The data should be provided as part of the manuscript or its supporting information, or deposited to a public repository. For example, in addition to summary statistics, the data points behind means, medians and variance measures should be available. If there are restrictions on publicly sharing data—e.g. participant privacy or use of data from a third party—those must be specified.

Reviewer #3: Yes

5. Is the manuscript presented in an intelligible fashion and written in standard English?

PLOS ONE does not copyedit accepted manuscripts, so the language in submitted articles must be clear, correct, and unambiguous. Any typographical or grammatical errors should be corrected at revision, so please note any specific errors here.

Reviewer #3: Yes

6. Review Comments to the Author

Please use the space provided to explain your answers to the questions above. You may also include additional comments for the author, including concerns about dual publication, research ethics, or publication ethics. (Please upload your review as an attachment if it exceeds 20,000 characters)

Reviewer #3: Methods

1. The data should be better explained to give the reader confidence about the categorization of some of the variables. For example, less than 3 previous births as the reference category – what about women without a previous pregnancy? It is not clear whether these are included in the reference category (and why this would be reasonable to do).

**We thank the reviewer for this suggestion - we have revised the description and also revised the categorization of the variable prior births and updated the analyses to reflect these changes.**

2. Some concerns around the inclusion of variables in the adjusted model if their association with the outcome was associated with p-value <= 0.15.

The authors should include all covariates in the adjusted model which are known risk factors for anemia. Results presented for the regression analysis seem to consider all covariates in the adjusted model despite the authors stating only covariates deemed significant from the univariate analysis would be considered. Further justification for the inclusion of covariates in the model should be provided.

**We agree with the reviewer’s observation. We believe that our original phrasing was confusing—we had intended to indicate that the primary consideration for inclusion in the model was theoretical justification, but in preliminary analyses we had also considered statistical significance. As we agree that our original description was confusing; we have revised the manuscript to more clearly indicate that justification for variable inclusion in the multiple logistic model was based on theoretical grounds.**

3. In the analytical plan, “complex survey design” was accounted for in the regression analysis but it is unclear what this means. The authors should provide further explanation.

**We have included additional detail and explanation as requested.**

Results

4. In the results section, it would be clearer to provide the percent (proportion) when reporting summary statistics from Table 1. Otherwise, the reader has to check these proportions themselves.

**Thank you – we have revised our presentation of the results of our study to reflect this recommendation.**

5. Figure resolution needs to be improved on all figures.

**We thank the reviewer for this observation – we have revised all of the figures to enhance their resolution. We have also revised the figure presenting the predicted probabilities, which we believe now provides an elegant, concise presentation of these results.**

Discussion

6. Reference to paper on childhood anemia by the authors and a comparison between the results of this study and the childhood anemia study would be helpful for conceptualizing this research.

**We thank the reviewer for this recommendation and their welcomed familiarity of our recently published study. We have included information from this study in our discussion section as suggested.**

7. Authors should provide more details on the limitations of the analysis - particularly on the categorization of some of the variables. This would give the reader more confidence that these limitations were considered in the interpretation of results.

**We have added additional details regarding limitations – though we believe some of the concern raised in this comment we have addressed through alternative coding for some of our variables.**

7. PLOS authors have the option to publish the peer review history of their article (what does this mean?). If published, this will include your full peer review and any attached files.

If you choose “no”, your identity will remain anonymous but your review may still be made public.

Do you want your identity to be public for this peer review? For information about this choice, including consent withdrawal, please see our Privacy Policy.

Reviewer #3: No

[NOTE: If reviewer comments were submitted as an attachment file, they will be attached to this email and accessible via the submission site. Please log into your account, locate the manuscript record, and check for the action link "View Attachments". If this link does not appear, there are no attachment files.]

While revising your submission, please upload your figure files to the Preflight Analysis and Conversion Engine (PACE) digital diagnostic tool, https://pacev2.apexcovantage.com/. PACE helps ensure that figures meet PLOS requirements. To use PACE, you must first register as a user. Registration is free. Then, login and navigate to the UPLOAD tab, where you will find detailed instructions on how to use the tool. If you encounter any issues or have any questions when using PACE, please email PLOS at figures@plos.org. Please note that Supporting Information files do not need this step.
